# Cell-based cccDNA reporter assay combined with functional genomics identifies YBX1 as HBV cccDNA host factor and antiviral candidate target

**DOI:** 10.1136/gutjnl-2020-323665

**Published:** 2022-12-09

**Authors:** Eloi R Verrier, Gaëtan Ligat, Laura Heydmann, Katharina Doernbrack, Julija Miller, Anne Maglott-Roth, Frank Jühling, Houssein El Saghire, Margaux J Heuschkel, Naoto Fujiwara, Sen-Yung Hsieh, Yujin Hoshida, David E Root, Emanuele Felli, Patrick Pessaux, Atish Mukherji, Laurent Mailly, Catherine Schuster, Laurent Brino, Michael Nassal, Thomas F. Baumert

**Affiliations:** 1 Université de Strasbourg, Inserm, Institut de Recherche sur les Maladies Virales et Hépatiques UMRS 1110, Strasbourg, France; 2 Department of Internal Medicine II/Molecular Biology, University Hospital Freiburg, Freiburg, Germany; 3 IGBMC, Plateforme de Criblage Haut-débit, Illkirch, France; 4 Department of Internal Medicine, Liver Tumor Translational Research Program, Simmons Comprehensive Cancer Center, Division of Digestive and Liver Diseases, University of Texas Southwestern Medical Center, Dallas, Texas, USA; 5 Department of Gastroenterology and Hepatology, Chang Gung Memorial Hospital, Taoyuan, Taiwan; 6 Broad Institute of Massachusetts Institute of Technology and Harvard, Cambridge, Massachusetts, USA; 7 Institut Hospitalo-Universitaire, Pôle Hépato-digestif, Nouvel Hôpital Civil, Strasbourg, France

**Keywords:** hepatitis B, antiviral therapy

## Abstract

**Objectives:**

Chronic hepatitis B virus (HBV) infection is a leading cause of liver disease and hepatocellular carcinoma. A key feature of HBV replication is the synthesis of the covalently close circular (ccc)DNA, not targeted by current treatments and whose elimination would be crucial for viral cure. To date, little is known about cccDNA formation. One major challenge to address this urgent question is the absence of robust models for the study of cccDNA biology.

**Design:**

We established a cell-based HBV cccDNA reporter assay and performed a loss-of-function screen targeting 239 genes encoding the human DNA damage response machinery.

**Results:**

Overcoming the limitations of current models, the reporter assay enables to quantity cccDNA levels using a robust ELISA as a readout. A loss-of-function screen identified 27 candidate cccDNA host factors, including Y box binding protein 1 (YBX1), a DNA binding protein regulating transcription and translation. Validation studies in authentic infection models revealed a robust decrease in HBV cccDNA levels following silencing, providing proof-of-concept for the importance of YBX1 in the early steps of the HBV life cycle. In patients, *YBX1* expression robustly correlates with both HBV load and liver disease progression.

**Conclusion:**

Our cell-based reporter assay enables the discovery of HBV cccDNA host factors including YBX1 and is suitable for the characterisation of cccDNA-related host factors, antiviral targets and compounds.

**WHAT IS ALREADY KNOWN ON THIS TOPIC**

- Antiviral therapeutic strategies can control hepatitis B virus (HBV) infection, but viral cure is rarely observed.
- Current antiviral therapies do not eliminate covalently close circular (ccc)DNA, HBV’s persistence reservoir.
- Knowledge about cccDNA formation and its host-dependency factors is limited.
- DNA repair factors are expected to play a central role in cccDNA formation.

**WHAT THIS STUDY ADDS**

- A cell-based reporter assay allows simple and robust quantification of cccDNA by ELISA.
- The assay enables discovery of HBV cccDNA host factors.
- A loss-of-function screen identified YBX1 as a previously undiscovered cccDNA-related HBV host factor that directly interacts with HBV DNA.
- Silencing of *YBX1* expression impairs HBV infection.
- Gene expression analyses in patients suggest an association between YBX1 and virus-induced liver disease and cancer.

**HOW THIS STUDY MIGHT AFFECT RESEARCH, PRACTICE OR POLICY**

- The cell-based cccDNA reporter assay will enable to discover urgently needed targets for viral cure in patients.

## Introduction

Chronic infection by hepatitis B virus (HBV) is a major cause of advanced liver disease and hepatocellular carcinoma (HCC), the second-leading cause of cancer death worldwide.^1^ Despite the availability of an effective prophylactic vaccine, an estimated 250 million people worldwide are chronic virus carriers.^2^ While nucleos(t)ide analogues (such as tenofovir or entecavir) have been proven to effectively control HBV infection by suppression of viral replication, treatment is lifelong and viral cure remains extremely rare. PEG-IFN-α-based therapies can result in viral cure in a small subset of patients; however, these therapies are limited by a low response rate and significant side effects.^3^

HBV belongs to the *Hepadnaviridae* family, small enveloped retrotranscribing DNA viruses.^4^ In infectious virions the genome is present as a 3.2 kb relaxed circular (rc) DNA in which one strand is covalently linked to the viral polymerase.^5^ Following entry into hepatocytes through heparansulfate proteoglycan and the liver-specific sodium-taurocholate cotransporting polypeptide (NTCP) as high affinity receptor,^6^ the nucleocapsid transports the rcDNA to the nuclear pore for release into the nucleus. There, rcDNA is converted into an episomal covalently close circular (ccc)DNA minichromosome that serves as a template for all viral transcripts including pregenomic (pg) RNA,^7^ which is encapsidated and reverse transcribed into new rcDNA. The rcDNA-containing nucleocapsids can be enveloped and released as virions or be recycled to the nucleus to replenish the cccDNA pool.^8^ Hence cccDNA formation is key to HBV persistence.^7^ A few cccDNA copies per liver cell can reactivate full virus production on therapy withdrawal, or on loss of immunological control over the low-level replication likely going on even under therapy. Any true cure of chronic HBV infection will require elimination of cccDNA.^7^

To date, the molecular basis of rcDNA to cccDNA conversion is poorly understood, largely due to the historic lack of feasible *in vitro* infection systems in which virus production is strictly dependent on cccDNA. Conceptual clues on the mechanism governing cccDNA formation come from the structural differences between the two DNAs. Accordingly, conversion comprises multiple steps, such as the release of the polymerase, trimming/fill-in of the DNA-strands, and eventually ligation. Given HBV’s minimal coding capacity, the underlying activities are likely to come from the host cell, in particular the DNA repair machinery, a network of ~250 factors evolved to fix any deviation from a perfect double-stranded structure.^7^ Based on this premise several recent studies unravelled the involvement of DNA repair factors in cccDNA formation. Tyrosyl-DNA-phosphodiesterase 2 (TDP2) was shown to be capable of releasing the bound polymerase from HBV and duck HBV (DHBV) rcDNA.^9^ A DNA polymerase-targeted siRNA screen identified DNA polymerase K (POLK), a gamma-family DNA polymerase, as a key contributor to the completion of the positive strand for the conversion of deproteinised (DP-) rcDNA into cccDNA.^10^ Flap endonuclease 1 (FEN1) has been shown to bind and cleave the 5’-flap structure of HBV rcDNA *in vitro* to promote cccDNA formation,^11^ possibly representing an alternative, nucleolytic pathway towards DP-rcDNA. Most recently, proliferating cell nuclear antigen (PCNA), replication factor C complex, DNA polymerase δ (POLD), FEN-1 plus DNA ligase 1 (LIG1) were found as the minimal host factor set for *in vitro* conversion of rcDNA-like molecules into cccDNA forms.^12^ However, the redundancies in the DNA repair machinery and even more the complex environment of an intact cell, including intracellular trafficking and metabolic turnover of the viral components, imply that cccDNA formation *in vivo* involves numerous additional host factors. Their identification will be crucial for a comprehensive understanding of HBV persistence as well the definition of new antiviral targets as a basis for a cure for chronic HBV infection; given their fundamental role in cellular DNA replication and apparent lack of HBV specificity the five minimally required already identified host factors are unlikely to make such targets.

In spite of the development of recent cell culture infection systems for HBV, a major limitation for the investigation of cccDNA formation is the limited number of cccDNA copies in infected cells.^13^ Moreover, cccDNA quantification by qPCR is still not finally standardised while Southern blotting, which allows for unambiguous distinction of cccDNA from all other viral DNA forms, is an intrinsically insensitive method.^13^ Hence, aiming to characterise novel cccDNA-related host factors we established in this study a novel cccDNA reporter cell line based on the >20 fold higher cccDNA copy numbers produced by DHBV in human HepG2 hepatoma cells.^8^ Applying to this cell line a loss-of-function screening strategy using an shRNA library targeting the DNA repair machinery and subsequent validation in HBV infection models we uncover YBX1 as a new cccDNA-related HBV host factor.

## Results

### A robust cell-based reporter assay models cccDNA formation using a simple ELISA-based readout for cccDNA quantification

**Figure 1:**
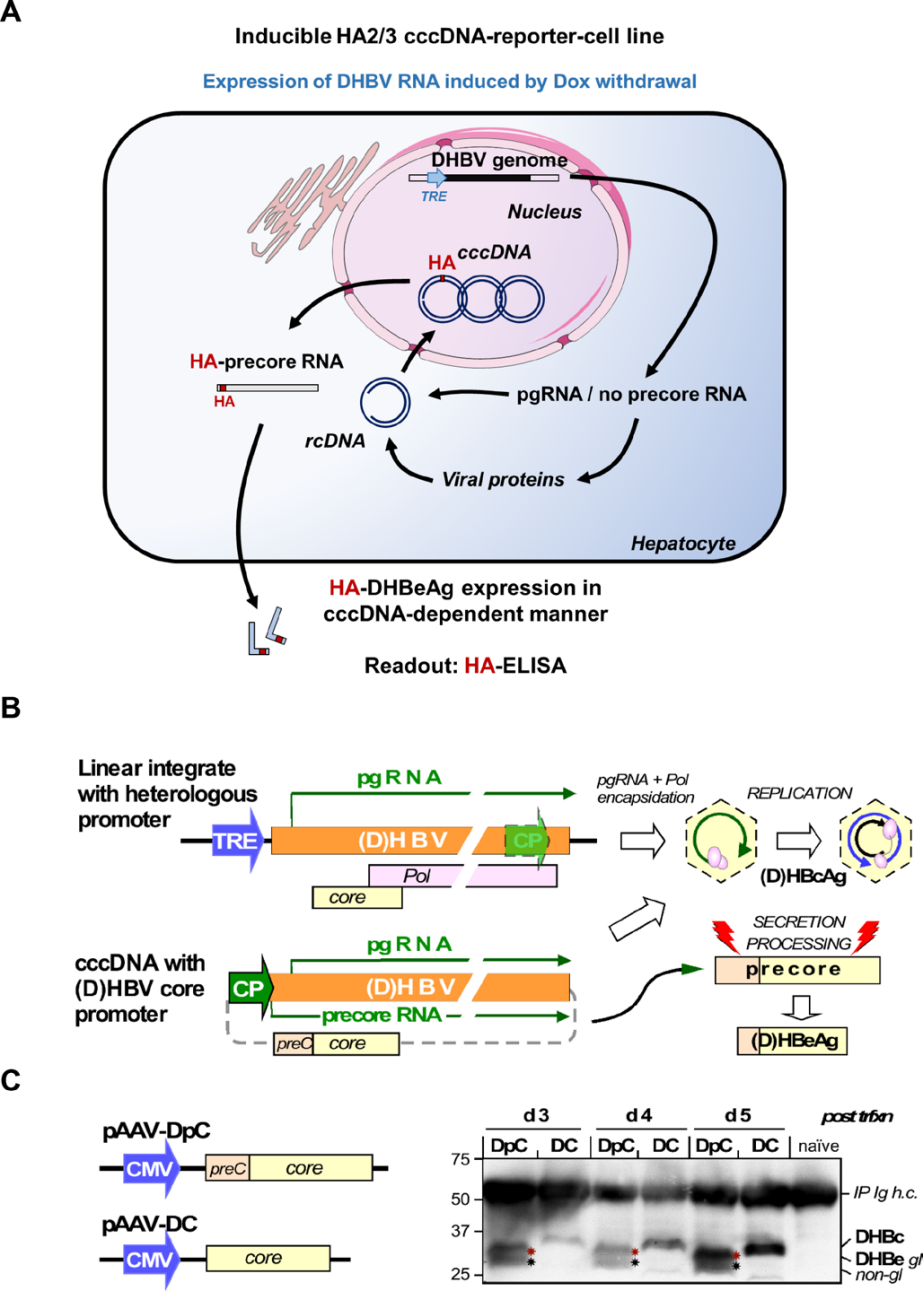
Use of secreted wild-type DHBeAg as non-invasive surrogate marker for cccDNA is frustrated by cellular release of DHBcAg. (A) Cartoon of the Tet-controlled HepG2 cell line expressing a modified full-length DHBV genome (with pgRNA expression under control of the Tet-responsive TRE promoter). Cells start producing DHBV RNA after removal of doxycycline (Dox) from the culture medium. expression of the DHBV envelope protein L is prevented by a premature stop codon. The cells synthesise no precore RNA whose start site would lie upstream of the start site defined by the TRE promoter. After pgRNA reverse transcription into DNA, DHBV DNA is recycled in the nucleus leading to the formation of cccDNA, which will serve as an additional template for viral transcription generating precore RNA and DHBV HBeAg (DHBeAg). Consequently, in this model, DHBeAg is a direct marker for cccDNA activity. In addition, a HA-tag encoding sequence was inserted into the precore region, before the start of the core ORF, leading to the production of a HA-tagged DHBeAg. (B) Assay principle. as mRNA for core protein and polymerase, and as substrate for reverse transcription pgRNA is sufficient to initiate hepadnaviral replication. Transcription of pgRNA from the authentic core promoter (CP) on cccDNA can be substituted by a heterologous promoter (eg, a Tet responsive TRE promoter) in front of a linear (D) HBV genome integrated into a plasmid or host chromosome. In this way no precore RNA and hence neither precore protein nor its prcessing product (D) HBeAg are generated (top). On circular cccDNA, the CP promoter controls pgRNA plus precore RNA transcription, supporting replication and (D) HBeAg synthesis (bottom). (C) Release of DHBcAg from cells obscures specific detection of DHBeAg. To mimic the translation products of pgRNA and precore RNA the ORFs for DHBV core (DC) and precore (DpC) proteins were expressed in transfected HepG2 cells from CMV promoter controlled pAAV vectors (left). Products in the culture supernatants were enriched by immunoprecipitation with a polyclonal anti-DHBc/e antiserum on days 3, 4 and 5 post-transfection and analysed by Western blotting. The DPC vectors produced a double band at about 27 and 30 kDa, as expected for non-glycosylated (non-gl, black stars) and glycosylated (gl, red stars) DHBeAg from DHBV16; clearly, however, also the DC vectors generated a specific band at about 30 kDa, as expected for DHBV core protein. Hence as reported for HBV,^19^ also the core protein from DHBV can be released from cells, likely as non-enveloped capsids. Hence in the absence of a truly preC specific antibody the presence of DHBcAg prevents unambiguous detection of DHBeAg in an ELISA format, ablating the cccDNA dependency of the assay. Specificity can be regained by selective tagging of DHBeAg (see figure 2). cccDNA, covalently close circular DNA; CMV, cytomegalovirus; DHBV, duck hepatitis B virus; IP Ig h.c., heavy chain of the immunoglobulin used for immunoprecipitation.

**Figure 2:**
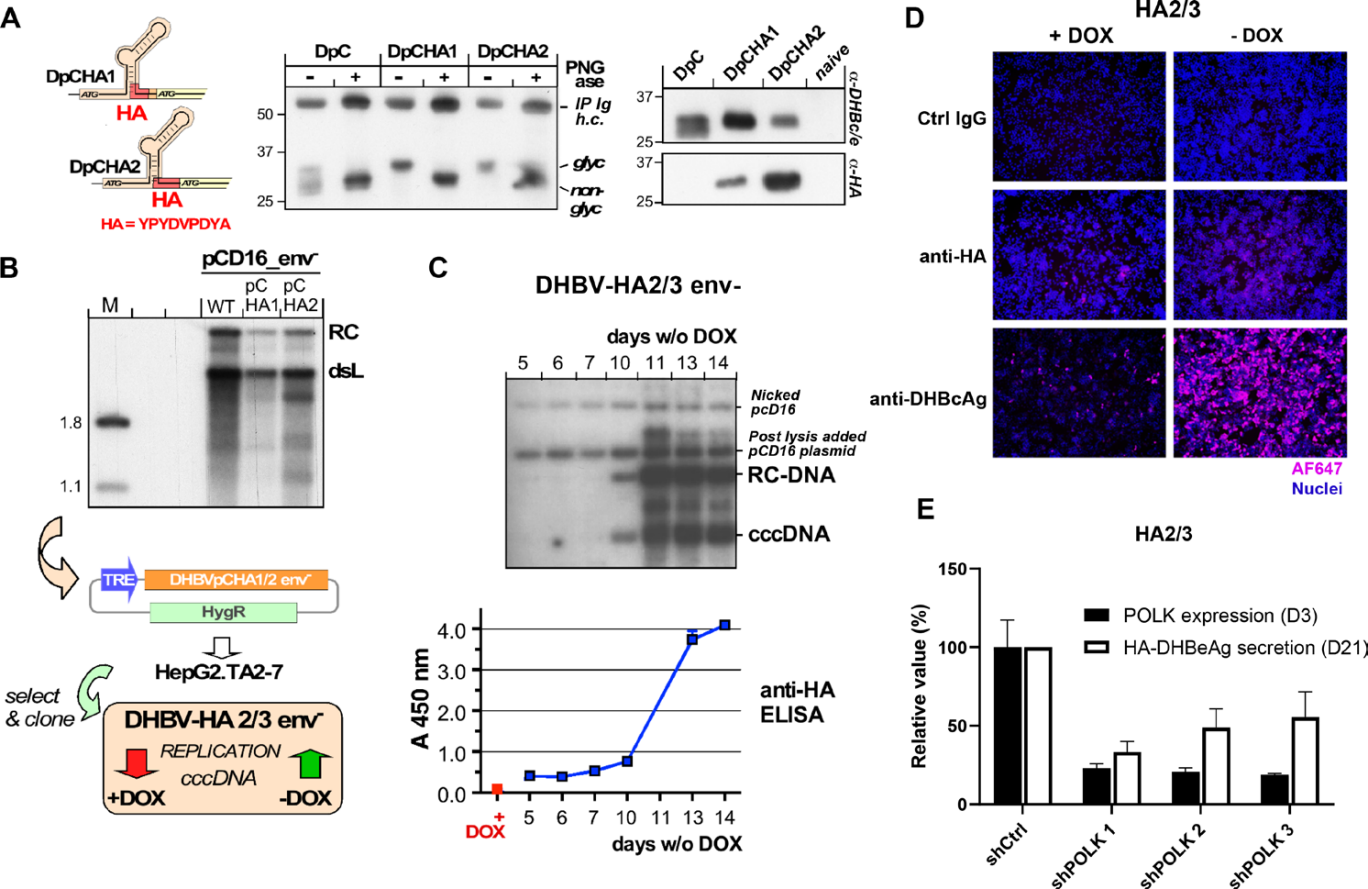
Strategically positioned HA-tags compatible with DHBV precore processing and replication. (A) glycosylation and retention of the HA-tag indicate DHBe-like secretion. constructs DpCHA1 and DpCHA2 differ in the insertion position of the HA-tag coding sequence in preC which is four amino acids upstream of the core ORF in DpCHA1, and immediately in front of the core ORF in DpCHA2 (left); the predicted signal sequence cleavage site (SignalP V3) is eight residues upstream. Vector pAAV-DpC and the HA-tag derivatives were transfected into HepG2 cells, and DHBc/e-reactive products in the culture supernatants were enriched by immunoprecipitiation. About one half of the immunopellet was treated with PNGase, the other half was not, then both were analysed by immunoblotting with polyclonal anti-DHBc/e antiserum (middle). While the doublet character of the main products from both HA-tag constructs was less pronounced than for the DPC construct, PNGase caused a similar mobility increase, indicating loss of glycosylation. Only the products from the HA-constructs were detected by a monoclonal anti-HA antibody (right), confirming that the secreted products contained the HA tag. (B) Replication competence. HepG2 cells were transfected with vector pCD16_env- encoding an envelope-deficient variant of wild-type DHBV16 (WT) and the respective pCHA1 and pCHA2 derivatives. Five days post-transfection viral DNAs from cytoplasmic capsids were analysed by southern blotting using a 32P-labelled DHBV DNA probe. although signals appeared slightly weaker than those from WT, both PchA derivatives clearly supported dsL and rc-DNA formation. The pCHA-modified DHBV genomes were recloned into vector pTRE2-hyg under control of the TRE promoter. The vectors were transfected into tet-transactivator 2 (tTA2) containing HepG2.TA2-7 cells, and individual cell clones were selected using hygromycin. Clone DHBV-HA2/3_env- showed good inducibility of DHBV replication on Dox withdrawal and was used for all further experiments. (C) Kinetics of cccDNA formation in induced DHBV-HA2/3 cells. After the indicated times in Dox-free medium (6-well format) cells were harvested and nuclear viral DNAs extracted using the QIAamp DNA mini kit were subjected to southern blotting as in (B); postnuclear lysis each sample was supplemented with 150 pg pCD16 plasmid as recovery control. maintenance of the HA sequence in cccDNA was confirmed by sequencing of the PCR amplicon generated from nuclear DNA using DHBV primers D26832+ (corresponding to DHBV16 positions 2178–2200) / D26814- (complementary to positions 71–95). In parallel, duplicate aliquots (200 µL each) from the culture supernatants of the cells were examined by sandwich ELISA using anti-DHBc/e coated plates plus a rat monoclonal anti-HA antibody - peroxidase conjugate (Roche #12013819001) for detection. (D) Detection of HA-DHBeAg and DHBcAg by if 19 days after Dox withdrawal in HA2/3 cells using specific antibodies. Nuclei were stained with DAPI. (E) Effect of *POLK* silencing on HA-DHBeAg secretion in HA2/3 cells. HA2/3 cells were plated in absence of Dox and transduced with lentivirus encoding three different shRNAs targeting Polk. 3 days after transduction, cells were lysed, total RNA was extracted and *POLK* expression was quantified by RT-qPCR. Results are expressed as means±SD% relative *POLK* expression compared with shCtrl (set at 100%) from two biological replicates. In parallel, supernatant from POLK-silenced cells were harvested 21 days post Dox withdrawal and HA-DHBeAg secretion was quantified by anti-HA ELISA. Results are expressed as means±SD% relative HA-HBeAg secretion compared with shCtrl (set at 100%) from four independent experiments. cccDNA, covalently close circular DNA; DHBV, duck hepatitis B virus; HA, haemagglutinin.

To address the limitations of detection of low abundance of cccDNA for functional genomics, we established a cell-based cccDNA reporter assay (figure 1A), which enables the non-invasive monitoring of cccDNA through a simple and robustly quantifiable ELISA for the cccDNA-dependent generation of a secreted, haemagglutinin (HA) tagged viral antigen in a stable HepG2 cell line, termed HA2/3. One key feature to achieve robust cccDNA levels in this model is the use of an envelope-deficient DHBV genome which can boost cccDNA copy numbers per cell to>100 in avian yet also in human hepatoma cells.^8^ In a first approach, an expression cassette wherein pgRNA transcription is controlled by a tetracycline (Tet) Response-Element (TRE) promoter was integrated into HepG2.TA2-7 cells^14^ which stably express a Tet-transactivator (tTA); the tTA binds to and activates the TRE promoter only in the absence of Tet or its analogue doxycyclin (Dox). Hence Dox withdrawal from the culture medium induces pgRNA transcription. Owing to its dual function as mRNA for core protein (HBcAg for HBV, DHBcAg for DHBV) and polymerase and as substrate for reverse transcription into rcDNA this is sufficient to initiate intracellular replication. If cccDNA is formed from the rcDNA, pgRNA transcription comes under control of the authentic viral core promoter which, different from the TRE promoter, produces an additional, 5’ extended transcript, the precore RNA (see figure 1A–C); this enables synthesis of the N terminally extended precore protein, precursor of the processed, secretory e antigen (HBeAg for HBV, DHBeAg for DHBV). Hence specific detection of eAg would indicate prior cccDNA formation, as previously proposed.^15^ It is well established that HBcAg and HBeAg share largely identical primary sequences but adopt different three-dimensional structures.^16 17^ Their serological discrimination relies largely on inaccessibility of epitopes in the assembled capsid and the scarcity of free capsids in serum, owing to immune complex formation. As this does not hold in cell culture, the naked HBV capsids known to be released from the cells,^18^ especially when damaged, can mock the presence of HBeAg. Via side-by-side expression of HBV core and precore protein we demonstrated that this is as well an issue with DHBV since DHBcAg accumulated to comparable amounts in the culture supernatant as the glycosylated and non-glycosylated forms of DHBeAg (figure 2A). Hence, DHBeAg could only serve as surrogate marker for cccDNA if strictly DHBeAg-specific antibodies were available which they are not.

We, therefore, sought to introduce a tag into the preC sequence such that it is exclusively expressed as part of the precore protein, does not interfere with secretion, and remains on DHBeAg on processing. Two constructs with a HA tag inserted shortly upstream of the core open reading frame (DpCHA1, DpCHA2) met these criteria, as shown by the secretion of glycosylated, anti-HA detectable proteins of the expected size (figure 2A). An important concern was the overlap on the pgRNA level of the preC coding region with the Dε RNA stem-loop that acts as encapsidation signal and replication origin for first-strand DNA synthesis; however, both constructs supported formation of wild-type like rc- and double-strand linear DNA (figure 2B) when part of the full DHBV genome in expression vector pCD16_env^-^. We therefore transfected an analogous TRE-promoter controlled DpCHA2 construct into HepG2.TA2-7 cells and, after evaluating various cell clones for cccDNA formation and HAeAg secretion, chose clone HA2/3 for all further experiments. Clonality of the cell line as well as efficient Dox regulation are supported by nearly all cells showing intracellular DHBcAg staining in the absence and very few cells staining positive in the presence of Dox. As shown in figure 2C, the accumulation of nuclear cccDNA around day 10 postinduction by Dox withdrawal was clearly reflected by a steep increase in HA signal intensity in the culture supernatant (monitored by a sandwich ELISA using a chromogenic substrate), attesting to secretion of HA-tagged DHBeAg as a valid surrogate marker for cccDNA formation. Furthermore, the very weak intracellular HA-staining suggests that the tagged DHBeAg is efficiently secreted (figure 2D).

Notably, the time it takes for cccDNA and DHBeAg to exceed the lower limit of detection correlates with the scale of the experiment; for instance, one well of a 6-well plate (as used here) holds about 20-fold more cells than one well of 96-well plate (as used in the shRNA screen). For the screening in 96-well format we therefore established a more sensitive chemiluminescent ELISA format whereby the increase in HA signal became evident from around day 14 to day 22 postinduction. As an adequately enduring silencing of host factors by chemical siRNAs did not appear feasible, we instead went for a lentiviral shRNA expression approach. Optimisation with the screening platform determined that D21 was an appropriate time point for the analysis of gene silencing impact on HA-DHBeAg. For validation, we transduced HA2/3 cells with lentiviruses encoding three different validated shRNAs targeting the expression of *POLK* which was previously described as an important contributor to the formation of HBV cccDNA,^10^ or a scrambled shRNA control. All three shRNAs achieved an approximately 80% reduction in *POLK* expression after 3 days (figure 2E), and this correlated with 40%–60% lower signals in the HA ELISA than in the shCtrl sample (figure 2E) 21 days after Dox withdrawal. Together these data indicated that the HA2/3 cell line is a relevant model to study cccDNA formation and regulation through the quantification of secreted DHBeAg, and hence is suited for the identification of cccDNA-related host factors through RNAi-based loss-of-function screening.

### A loss-of-function screen targeting the DNA damage response (DDR) machinery uncovers 27 candidate HBV cccDNA host-dependency factors

**Figure 3:**
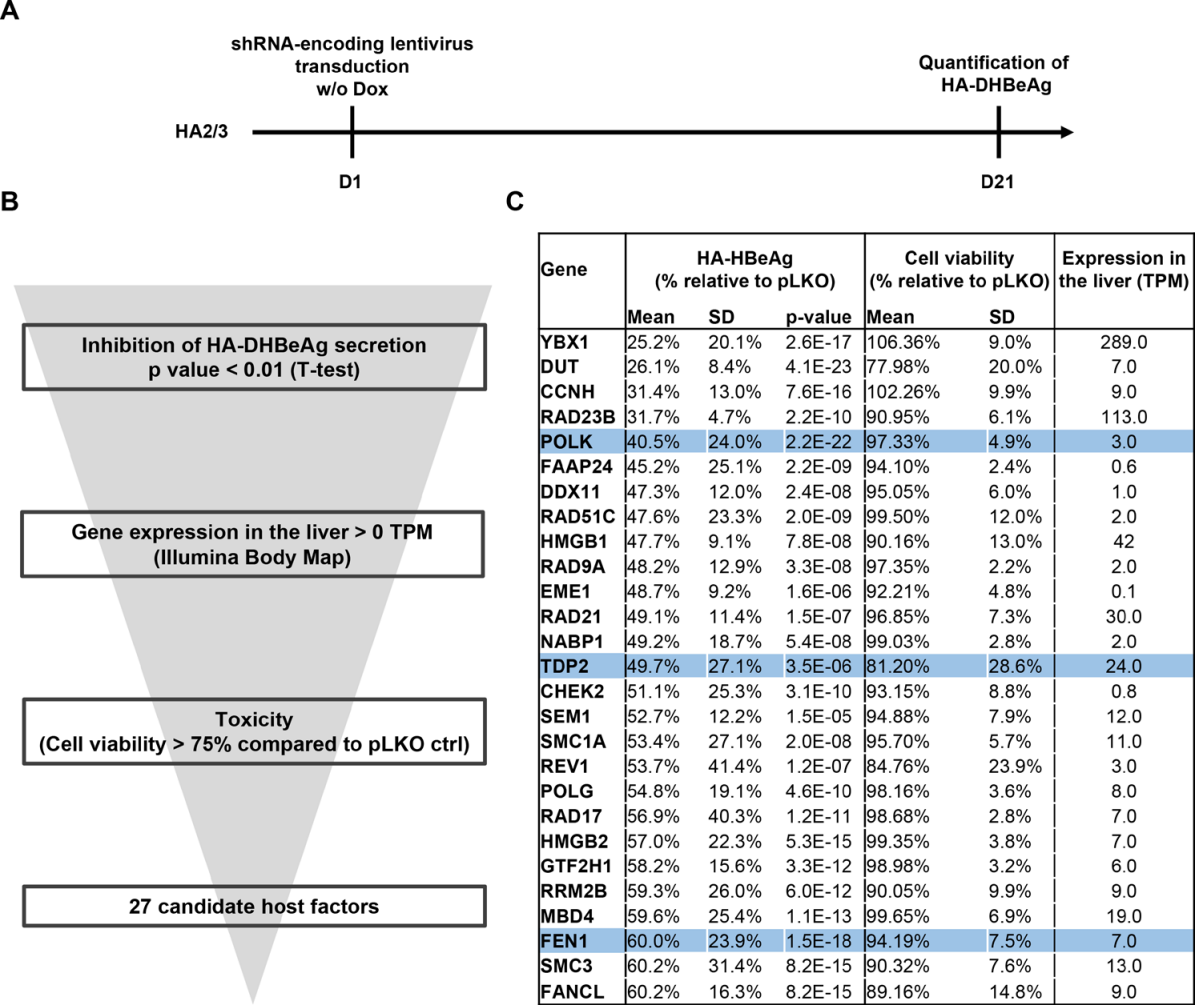
Approach of loss-of-function screen (LOF) in HA2/3 cells for the identification of HBV cccDNA host-dependancy factors. A. schematic workflow of LOF-screen. (B) Selection of candidate genes from the primary screen with the phenotypic robustness between replicates, the expression in the liver (illumina body MAP) and the toxicity (cell viability >75% compared with pLKO ctrl). (C) 27 candidates from the primary screen. The global effect of individual gene silencing on HBeAg production was analysed by pooling the effect of the three-independent shRNA per gene (n=9). P values were obtained through a two-tailed Student’s t-test comparing phenotypic effects of shRNAs and pLKO ctrl. *POLK*, *TDP2* and *FEN1* are highlighted in blue. cccDNA, covalently close circular DNA; FEN1, flap endonuclease 1; HA, haemagglutinin; HBV, hepatitis B virus.

Given the anticipated crucial role of DNA repair in cccDNA formation, we next devised a lentiviral shRNA library targeting 239 genes belonging to the DNA damage response (DDR) machinery, with each gene being targeted by three independent shRNAs (online supplemental table S1). shRNAs targeting *POLK* expression were additionally used as positive controls, while empty pLKO vector and turboGFP expression vectors were used as negative controls. We then applied this targeted shRNA screen to the HA2/3 cell line, using as readout the HA-tagged DHBeAg ELISA values at 21 days after doxycycline withdrawal (figure 3A). Long-term silencing efficacy induced by shRNA was assessed by quantification of gene expression for one target (online supplemental figure S1). Using this approach, we identified a series of HBV cccDNA host factor candidates in which shRNA transduction correlated with a significant decrease in HA-DHBeAg production (figure 3B and online supplemental table S2). Computational analysis of the candidates including phenotypic variability and significance between replicates, toxicity and expression in the liver resulted in a short list of 27 candidates. Importantly, they included *POLK*, *TDP2* and *FEN1*, previously described as cccDNA-related HBV host factors, supporting the validity of our approach to uncover cccDNA host-dependency factors (figure 3C). Among the top-scoring candidates was *YBX1*, encoding the Y box binding protein 1 (formerly YB-1), also known as nuclease-sensitive element-binding or Y-box transcription factor. YBX1 is a DNA/RNA binding protein involved in several cellular processes, including DNA repair, regulation of transcription and translation, pre-mRNA splicing and mRNA packaging, that is, formation of mRNA nucleoprotein complexes.^19^ Another top candidate was the DUT encoding deoxyuridine triphosphatase, a key enzyme of nucleotide metabolism which hydrolyzes dUTP to dUMP and pyrophosphate. DUT is an essential enzyme for maintaining DNA integrity by preventing misincorporation of uracil into DNA, which results in DNA toxicity and cell death.^20^ Interestingly, dUTPase enzymes are encoded by many retroviruses and were shown to be essential for viral replication of HIV types 1 and 2.^20^ As a pararetrovirus expressing no dUTPase, endogenous dUTPase may well play an important role in the life cycle of HBV by preserving the integrity of viral DNA. Yet another top-scorer was *DDX11* which encodes a DEAD box DNA helicase (DDX11) involved in DNA replication recovery from DNA damage.^21^ An early step in cccDNA conversion from rcDNA is the removal of protein P and the RNA oligomer from the 5’ ends of the two DNA strands. Removal of the respective flap-structures has been ascribed to FEN1 which is also one of the five core factors for *in vitro* cccDNA formation.^12^ DDX11 cooperates with FEN1 during removal of 5’-flap structures during Okazaki fragment maturation and long-patch base excision repair.^22^

10.1136/gutjnl-2020-323665.supp1 Supplementary data

10.1136/gutjnl-2020-323665.supp5 Supplementary data

10.1136/gutjnl-2020-323665.supp2 Supplementary data

Taken together, our approach enabled to uncover several previously unidentified cccDNA-related host factor candidates. Given the HBV infection phenotype on silencing, absent or at most limited host cell toxicity during silencing, high expression in the liver (figure 3C) and previously described targetability by small molecules^23 24^ we chose to focus on YBX1 for further analyses. In this context, we first confirmed by Southern blot the effect of YBX1 and POLK depletion on human HBV cccDNA levels in another reporter cell line, HAC18, which expresses the HBV genome in an analogous manner to the DHBV genome in HA2/3 cells (online supplemental figure S2).

### Validation of YBX1 as an HBV host factor in an HepG2-NTCP cell infection model

**Figure 4:**
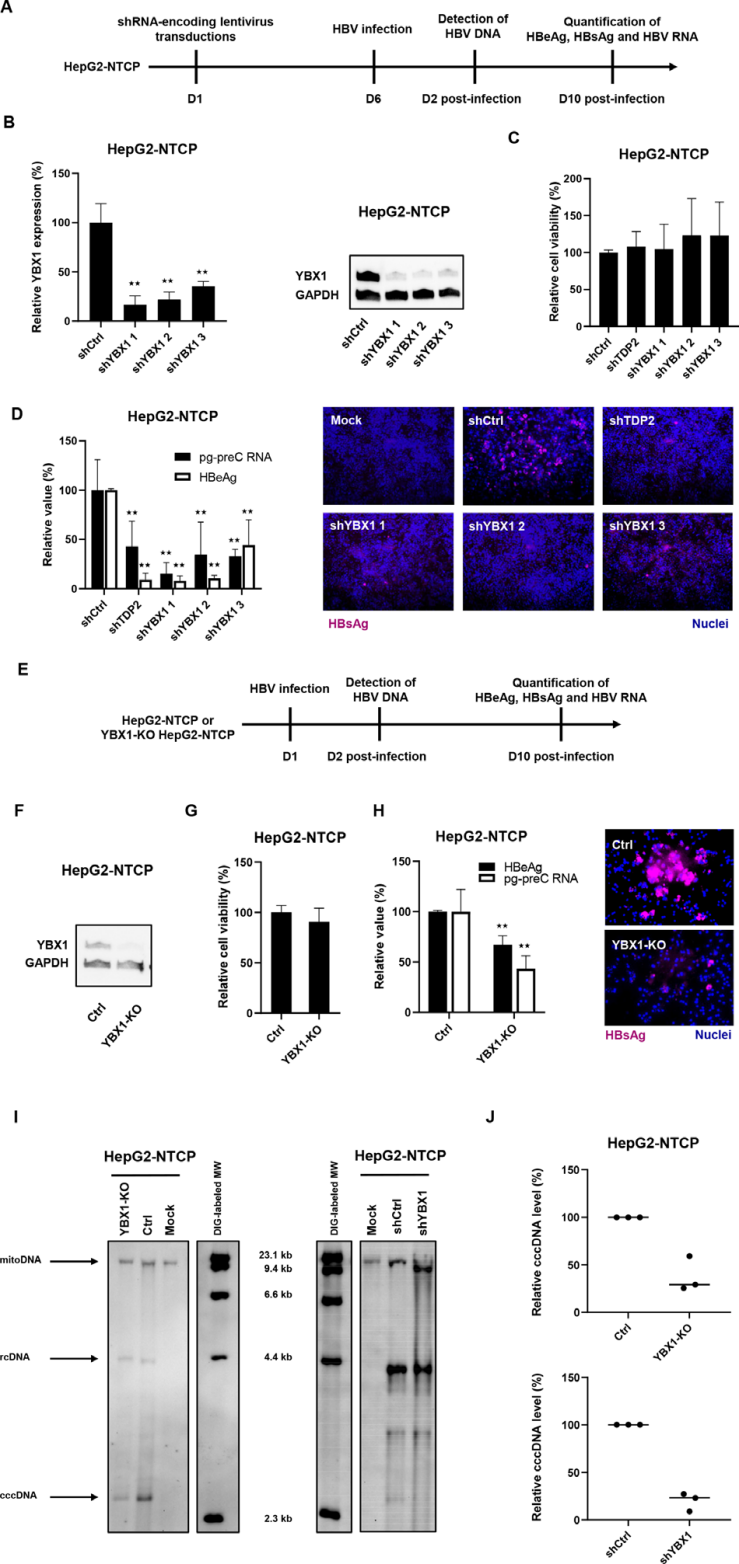
Validation of YBX1 as a HBV cccDNA host-dependency factor in the HBV permissive cell line HepG2-NTCP. (A) Experimental approach and timelines for the shRNA-based validation approach. HepG2-NTCP cells were transduced with lentivirus encoding three *YBX1*-targeting shRNA (shYBX1 1, 2, 3) 3 days post-transduction, total RNA was extracted and *YBX1* expression was quantified by RT-qPCR (B), left panel). Results are expressed as means±SD% relative *YBX1* expression compared with shCtrl (set at 100%) from three independent experiments. in parallel, *YBX1* expression was controlled by Western blot in transduced HepG2-NTCP (B), right panel). One representative blot is shown. 6 days post-transduction, cells were then infected with HBV. (C) Cell viability was assed 10 days postinfection (DPI). Results are expressed as means±SD% relative cell viability compared with shCtrl (set at 100%) from three independent experiments. (D) HBV infection was assessed by quantification of HBV markers at 10 DPI: secreted HBeAg by CLIA (white) and HBV pg-preC RNA by RT-qPCR (white). Results are expressed as means±SD% relative HBV infection compared with shCtrl (set as 100%) from three independent experiments. intracellular HBsAg expression was assessed by if 10 DPI. (E) Experimental approach and timelines for the CRISPR/Cas9 validation approach. *YBX1*-KO cell line was produced from HepG2-NTCP cells through CRISPR/Cas9 technology using a specific sgRNA targeting *YBX1*. (F) YBX1 protein expression in *YBX1*-KO cells compared with parental HepG2-NTCP cells (Ctrl) was assessed by Western blot. cells were then infected with HBV for 10 days G. cell viability was assessed 10 DPI. Results are expressed as means±SD% relative cell viability compared with HBV-infected HepG2-NTCP (set at 100%) from three independent experiments. (H) HBV infection was assessed 10 DPI by quantification of secreted HBeAg by CLIA (black) and HBV pg-preC RNA quantification by RT-qPCR (white). Results are expressed as means±SD% relative HBV infection compared with HBV-infected HepG2-NTCP (set as 100%) from three independent experiments. Intracellular HBsAg expression was assessed 10 dpi by if. (I) Detection of HBV DNAs by Southern blot from *YBX1*-silenced and *YBX1*-KO cells. HBV rcDNA and HBV cccDNA are indicated. One representative experiment is shown. (K) Quantification of cccDNA levels. cccDNA levels were quantified using ImageJ software. Three independent experiments are shown. ^★★^p<0.01 (two-tailed Mann-Whitney U test). cccDNA, covalently close circular DNA; HBV, hepatitis B virus; NTCP, sodium-taurocholate cotransporting polypeptide.

Next, we aimed to validate the role of YBX1 in state-of-the-art HBV infection models. Taking advantage of our previously described HepG2-NTCP cell line susceptible to robust HBV-infection,^25^ we performed silencing studies using shRNA targeting *YBX1* expression. HepG2-NTCP were individually transduced with three different shRNA-containing lentivirus constructs, selected with puromycin 48 hours prior to HBV infection and harvested 10 days postinfection (figure 4A). While *TDP2*-knock-out (KO) cells have been reported to be permissive to HBV infection,^26^ DHBV cccDNA accumulation was slowed down in these cells.^26^ Moreover, *TDP2* was identified as a target in our primary screen. Consequently, *TDP2*-targeting shRNA was used as a control. Knockdown of both *TDP2* and *YBX1* resulted in a marked reduction of secreted HBeAg, intracellular HBV RNA (both pgRNA and PreC RNA (see online supplemental table S3) and intracellular HBsAg levels (figure 4B–D), revealing a significant decrease in HBV infection in *YBX1*-silenced HepG2-NTCP cells.

10.1136/gutjnl-2020-323665.supp3 Supplementary data

We then generated *YBX1*-KO HepG2-NTCP cells using CRISPR-Cas9 technology (figure 4E) and specific guide RNA. A robust decrease in YBX1 expression was observed in cells transduced with sgRNA targeting *YBX1* (figure 4F). Functional analyses on infection with HBV showed a marked decrease in the levels of HBV markers in *YBX1*-KO HepG2-NTCP cells compared with HepG2-NTCP not related to cell viability (Figure 4G,H). Collectively, these functional analyses support an important role for YBX1 in HBV infection of HepG2-NTCP cells.

To study the functional role of YBX1 for the formation and/or maintenance of HBV cccDNA levels, we assessed HBV DNA replication intermediates in HBV infected *YBX1*-silenced HepG2-NTCP cells by Southern blot. As shown in figure 4I and figure 4J, shRNA-induced silencing of *YBX1* resulted in a marked decrease in cccDNA levels relative to non-silenced HBV-infected cells. The identity of the band marked as cccDNA was confirmed by heat denaturation and subsequent linearisation by EcoRI digestion (online supplemental figure S3). Similar results were observed in HBV-infected *YBX1*-KO HepG2-NTCP cells (Figure 4I,J). Interestingly, no decrease in HBV infection was observed when *YBX1* knockdown in HBV-infected HepG2-NTCP cells was achieved after virus infection when the initial cccDNA pool is already established (online supplemental figure S4). Comparable results were observed in HA2/3 cells (online supplemental figure S4).

Finally, we aimed to clarify the mode of action by which YBX1 affects the early steps of the HBV life cycle. First, loss-of-function studies did not reveal a significant modulation of key cccDNA host factor expression on *YBX1* knockdown, including *POLK*, *TDP2* and *PCNA* (online supplemental figure S5A). Next, we functionally excluded a major role of YBX1 on viral cell entry, as no effect on infection of *YBX1*-KO Huh7-NTCP cells by hepatitis D virus was observed (online supplemental figure S5B-D) which, like HBV, uses NTCP as entry receptor. Taken together, these results confirm a key functional role of YBX1 in early steps of the HBV life cycle in infected hepatocytes, subsequent to virus entry but prior to the establishment of the initial cccDNA pool.

**Figure 5:**
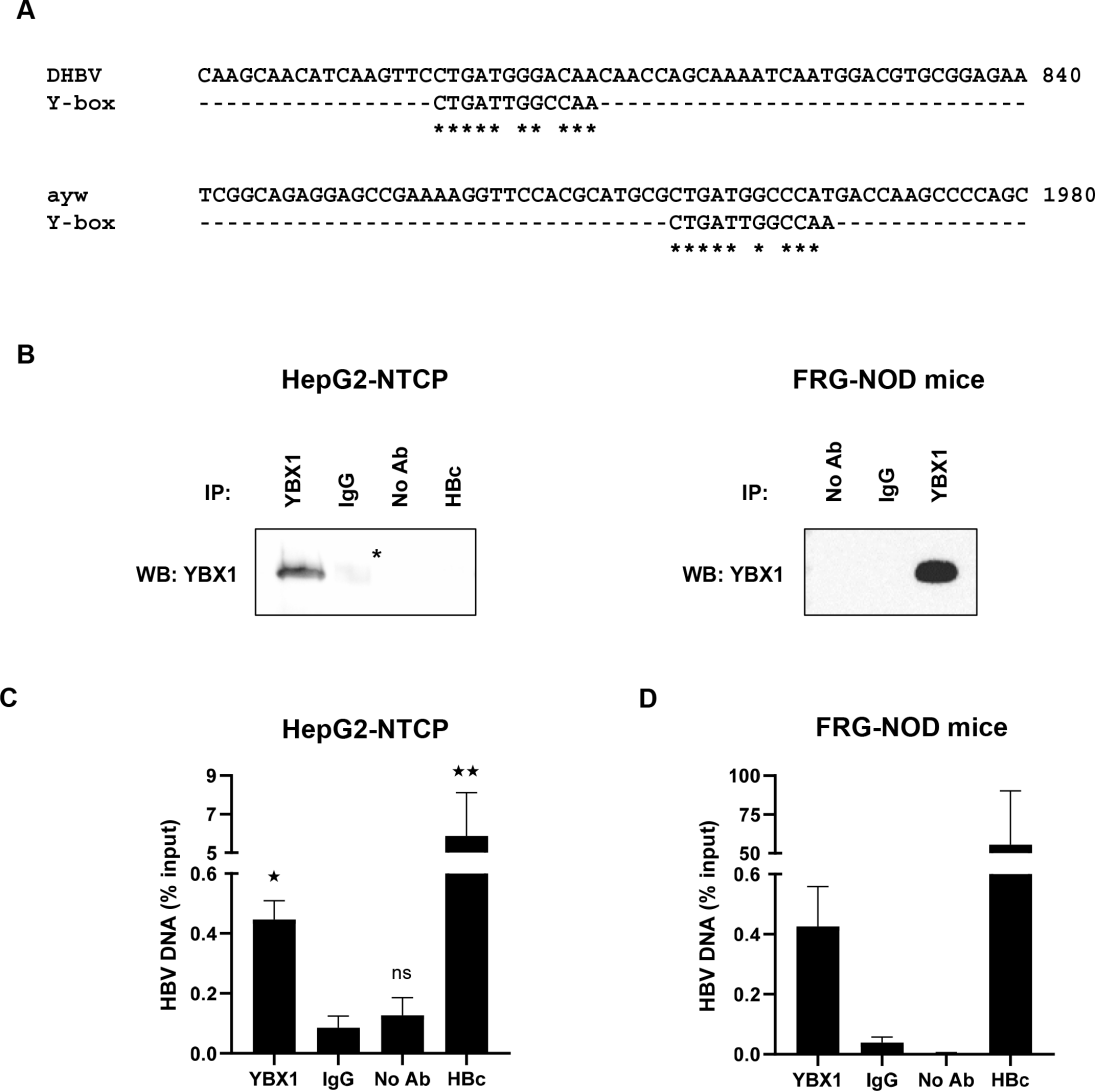
YBX1 binds to HBV DNA. (A) Putative Y-box motifs within the DHBV (K01834.1) and HBV genomes (ayw strain; V01460.1). (B, C). ChIP-qPCR assay on HBV-infected HepG2-NTCP cells. HepG2-NTCP cells were infected with HBV. nuclear DNA was extracted two DPI and CHIP was performed using a specific anti-YBX1 antibody and a specific anti-HBcAg (HBc) antibody as a positive control. Immunoprecipitation efficacy was assessed by Western blot (B). The StAR in the blot corresponds to non-specific signal due to the presence of heavy chains (around 50 kDa) from the rabbit isotype control used for the immunoprecipitation and that are still weakly detected despite the use of an adapted HRP reagent. HBV DNA in immunoprecipitated eluates was quantified by qPCR (C). Results are shown as means±SEM% HBV DNA in eluates compared with input samples from five biological replicates. (D) The same ChIP-qPCR assay was performed using nuclear extracts from HBV-infected FRG-NOD mice. Results are shown as means±SEM% HBV DNA in eluates compared with input samples from three independent mice (one sample per mouse). ^★^p<0.05; ^★★^p<0.01 (two-tailed Mann-Whitney U test). ChIP, chromatin immunoprecipitation; HBV, hepatitis B virus; NS, non-significant

### YBX1 is recruited to HBV DNA

The above results led us to hypothesise that due to its nucleic acid binding abilities,^19^ YBX1 might directly interact with the HBV genome to promote cccDNA formation and accumulation. YBX1 is well known to bind to promoter/enhancer regions containing a Y-box motif (5'-CTGATTGGCCAA-3'),^27^ although other DNA and RNA sequences can also be bound.^28^ Moreover, it has been previously reported that YBX1 can bind to integrated HBV DNA regions.^29^ Importantly, using sequence alignments as shown in figure 5A, we found putative Y-box motifs within the HBV ayw reference sequence (GenBank V01460.1) as well as within the DHBV16 reference sequence (GenBank K01834.1). Therefore, to investigate whether the YBX1-induced HBV regulation involves the recruitment of YBX1 to the HBV genome, we performed chromatin immunoprecipitation (ChIP) qPCR assays on nuclear extracts from HBV-infected HepG2-NTCP cells with an anti-YBX1 antibody and an anti-HBcAg antibody as a positive control (figure 5B). Immunoprecipitation efficacy was controlled by Western blot detection of the precipitated proteins (figure 5B). Importantly, ChIP-qPCR analysis revealed specific recruitment of YBX1 to HBV DNA in the nucleus of infected HepG2-NTCP cells (figure 5C). Notably, we confirmed this result using nuclear extracts from three HBV-infected chimeric FRG-NOD mice (online supplemental table S4) suggesting that this interaction is present during HBV infection *in vivo*, although the kinetics of this interaction in the context of cccDNA steady-state levels still remains to be determined, since the ChIP assays was performed at a single time point both in cell culture models and in chronically infected human chimeric mice (figure 5C,D).

10.1136/gutjnl-2020-323665.supp4 Supplementary data

### The rescue of YBX1 expression in KO cells restores cccDNA formation and HBV infection

**Figure 6:**
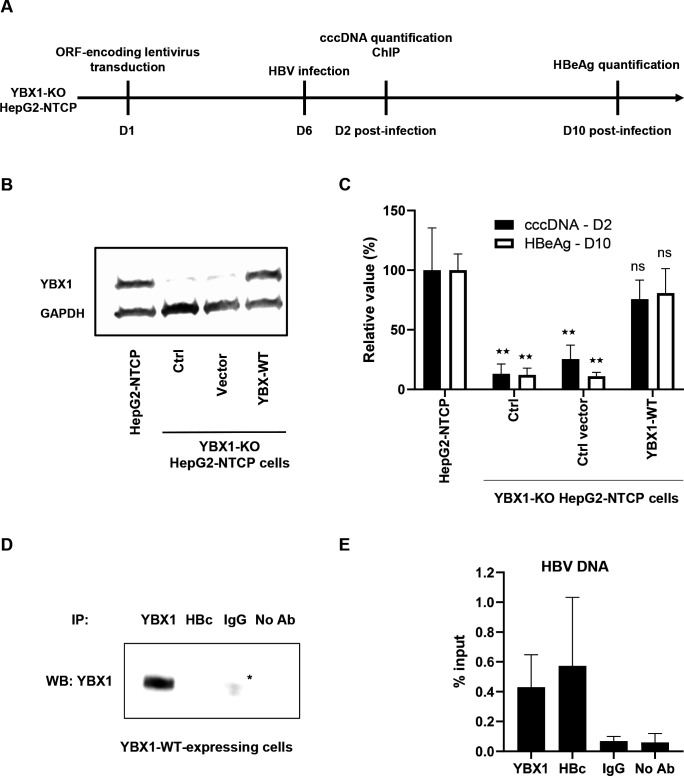
Rescue of YBX1 expression restores HBV infection in *YBX1*-KO cells. (A) Experimental timelines. *YBX1*-KO cells were transduced with lentivirus encoding the wild type version of YBX1 (YBX1-WT). (B) YBX1 expression was controlled by Western blot. 6 days after transduction, cells were infected by HBV. Two days postinfection, DNA was extracted and cccDNA levels were quantified by specific qPCR (C). Results are expressed as means±SD% relative cccDNA levels secretion compared with HepG2-NTCP cells (set as 100%) from three independent experiments. in parallel, secreted HBeAg levels were quantified 10 DPI by CLIA. Results are expressed as means±SD% relative HBeAg secretion compared with HepG2-NTCP cells (set as 100%) from three independent experiments (C–E). In parallel, ChIP-qPCR assay was performed on YBX1-WT-expressing HepG2-NTCP cells infected by HBV at day two postinfection using a specific anti-YBX1 antibody, a specific anti-HBc antibody, or a control IgG. YBX1 immunoprecipitation efficiency was assessed by Western blot (D). The StAR in the blots correspond to non-specific signal due to the presence of heavy chains (around 50 kDa) from the rabbit isotype control used for the immunoprecipitation (E). Results are expressed means±SDM HBV DNA levels (% input) from three biological replicates. ^★★^p<0.01 (two-tailed Mann-Whitney U test). cccDNA, covalently close circular DNA; ChIP, chromatin immunoprecipitation; HBV, hepatitis B virus; KO, knock-out; NS, non-significant; NTCP, sodium-taurocholate cotransporting polypeptide.

To validate the canonical association between YBX1 and HBV DNA, we took advantage of *YBX1*-KO HepG2-NTCP cells (figure 6A). We designed *YBX1* cDNA constructs carrying silent mutations in the sgRNA target sequence, and coding for the wild type amino acid sequence of YBX1 (YBX1-WT). We restored YBX1 expression in the *YBX1*-KO HepG2-NTCP cells, leading to robust and similar protein expression compared with endogenous levels (figure 6B). These cells were then infected by HBV. As shown in figure 6C, ectopic restoration of YBX1-WT was associated with a rescue of HBV infection phenotype, as measured by HBeAg secretion at day 10 postinfection. Importantly, YBX1-WT expression restored cccDNA levels at day 2 postinfection as shown by qPCR, confirming the importance of YBX1 in the early steps of the HBV life cycle and cccDNA formation or regulation (figure 6C). The increase in cccDNA levels in *YBX1*-KO cells expressing YBX1-WT was confirmed by Southern blot analyses (online supplemental figure S6). Finally, we performed ChIP-qPCR assays on nuclear extracts from HBV infected *YBX1*-KO cells expressing either YBX1-WT and confirmed the ability of the ectopic version of YBX1 to bind HBV DNA (figure 6E). Taken together, our results confirm the importance of YBX1 in the early steps of the viral life cycle and the early formation or regulation of cccDNA.

### Validation of functional role of YBX for the HBV life cycle in primary human hepatocytes

**Figure 7:**
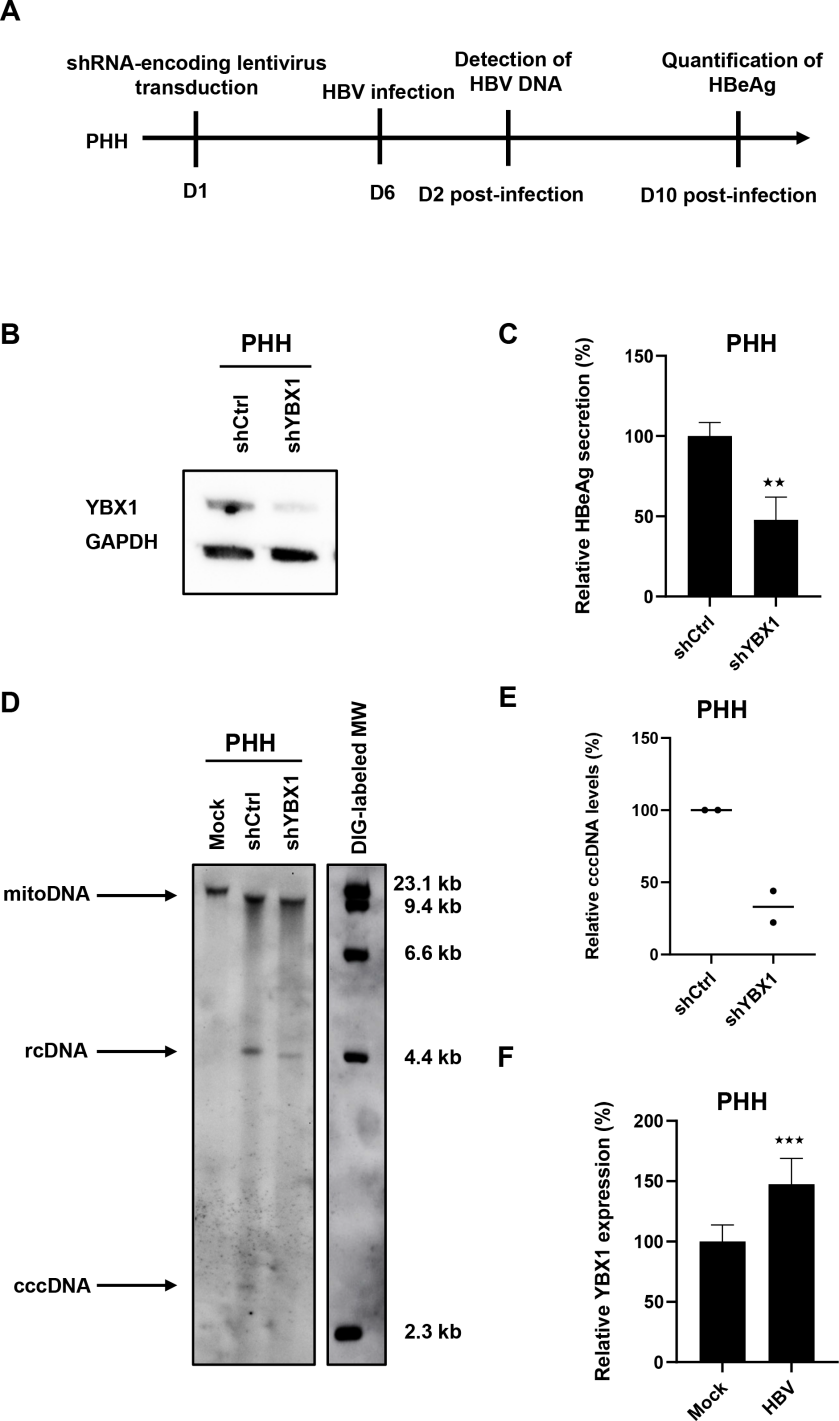
Ybx1 knock-down inhibits HBV infection in primary human hepatocytes. (A) Experimental approach and timelines. Primary human hepatocytes (PHH) cells were transduced with lentivirus encoding a *YBX1*-targeting shRNA (shYBX1) 3 days post-transduction, YBX1 expression was controlled by Western blot. (B) One representative blot is shown. Six days post-transduction, PHH cells were then infected with HBV, and HBV infection was assessed by quantification of secreted HBeAg by CLIA 10dpi. Results are expressed as means±SD% relative HBV infection compared with shCtrl (set as 100%) from three independent experiments. (C) In parallel, PHH cells were lysed at day 2 postinfection, DNA was extracted and HBV DNA was detected by Southern blot (D). HBV rcDNA and HBV cccDNA are indicated. One representative experiment is shown. (E) Quantification of cccDNA levels using ImageJ software from two independent experiments. (F) *YBX1* mRNA expression in HBV-infected PHH. PHH were infected with HBV and *YBX1* expression was assessed 10 dpi. Results are expressed as means±SD% relative *YBX1* expression compared with shCtrl (set at 100%) from four independent experiments. ^★★^ p<0.01; ^★★★^ p<0.01 (two-tailed Mann-Whitney U test). cccDNA, covalently close circular DNA; HBV, hepatitis B virus; RCdna, relaxed circular dna.

We next validated the functional role of YBX1 for the HBV life cycle in primary human hepatocytes, the *in vitro* model most closely related to HBV infection in patients (figure 7A). Silencing of *YBX1* expression in PHH prior to HBV resulted in a marked decrease in HBeAg secretion and cccDNA levels (figure 7B–E). These data confirm in the clinically most translatable HBV infection cell-based model a functional role of YBX1 in the HBV life cycle. Of note, *YBX1* expression was upregulated in HBV-infected PHH compared with non-infected cells (figure 7F).

### Analysis of *YBX1* gene expression in patients with chronic HBV infection and liver disease

Based on our observation that HBV can modulate *YBX1* expression in PHH (figure 7F), we next studied *YBX1* gene expression in liver tissues in different cohorts of patients with HBV infection and liver disease. As controls we used hepatitis C virus (HCV)-infected or NASH patients with a similar disease phenotype.

**Figure 8:**
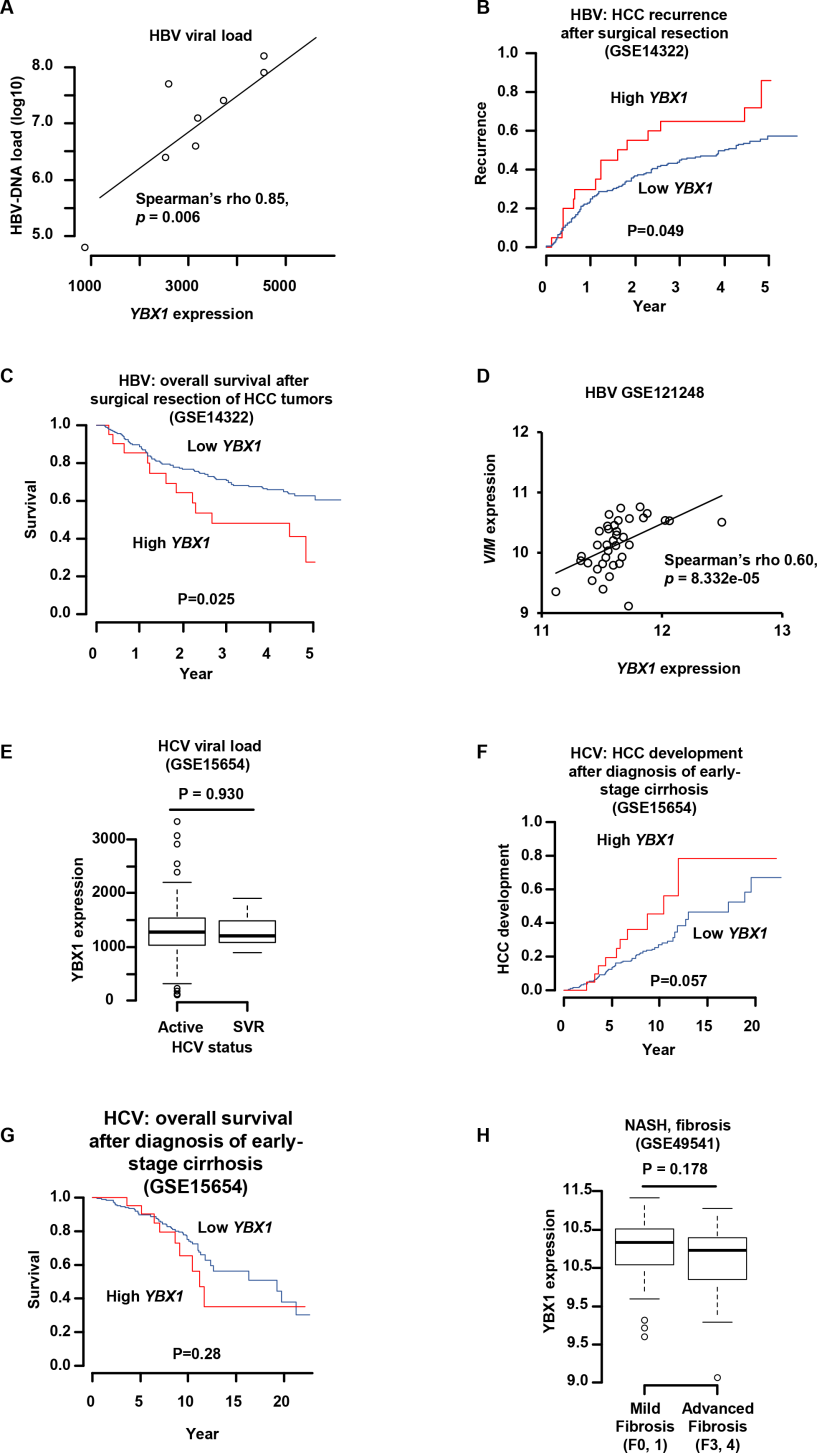
*YBX1* expression correlates with HBV load, HCC recurrence and survival in patients. (A) *YBX1* mRNA expression in HBV-infected patients (cohort described in methods). (B, C) Tumour recurrence (B) and survival (C) and level of *YBX1* expression (low-high) in a cohort of HBV-induced HCC patients (GSE14322). (D) Correlation between *YBX1* expression and *VIM* expression in HBV-infected patients (GSE121248). (E) Correlation between *YBX1* expression and the NASH fibrosis stage (GSE49541). (F) Correlation between *YBX1* expression and the HCV status. (G, H) Survival (G) and HCC development (H) depending on level of *YBX1* expression (low-high) (GSE15654). HBV, hepatitis B virus; HCC, hepatocellular carcinoma; HCV, hepatitis C virus.

A significant positive correlation (Spearman’s r=0.85, p=0.006) was observed between HBV viral load and *YBX1* expression in liver tissues from nine HBV-infected patients (GSE14322) (figure 8A),^25^ suggesting that YBX1 may be a clinically relevant factor for HBV infection.^30^ Indeed, higher expression of *YBX1* in the livers from patients with surgically resected HBV-related HCC was associated with significantly higher probability of tumour recurrence after the resection (figure 8B) and eventually lower long-term overall survival rate (figure 8C). Furthermore, we observed a marked and significant correlation between *YBX1* expression and the expression of fibrosis-associated genes, including VIM, encoding the vimentin in HBV-infected patients (GSE121248) (Spearman’s r=0.60/ p = 8.332e-05) (figure 8D). Vimentin is a well-described biomarker for several cancers including sarcoma,^31^ known to be involved in the progression of liver disease and HCC.^32^

In contrast, there were no significant associations with fibrosis progression in a cohort of NASH patients (GSE49541) or HCV load, although a non-significant positive tendency was observed between *YBX1* expression and disease progression and survival in patients with HCV-related cirrhosis (GSE15654) (figure 8E–H).

## Discussion

In this study, we established a novel cell-based reporter assay using a simple ELISA as a readout which enables fast-track discovery of HBV cccDNA host factors as antiviral targets for cure. Using this assay, we identified YBX1 as a previously undiscovered HBV host-dependency factor. Moreover, gene expression analyses in patients suggest that YBX1 is clinically not only relevant for the HBV life cycle but also for the pathogenesis of virus-induced liver disease and cancer.

A key limitation for the investigation of cccDNA formation is the low number of cccDNA copies in infected cells. The cell-based reporter system described in this study overcomes this challenge by enabling the non-invasive monitoring of cccDNA via the cccDNA-dependent generation of a secreted HA tagged DHBeAg. We note that Cai *et al* independently described a principally similar system for HBV.^33^ In our study, we decided to initially focus on DHBV, a hepadnavirus closely related to HBV, because it produces a much higher number of cccDNA copies per cell compared with human HBV even in the same human hepatoma cells.^8^ This approach can be scaled up to 96 or 384 well plates allowing medium-sized drug or loss-of-function screens. The ELISA format allows simple automatic or robotic analyses. The inter-assay reproducibility is high, thus minimising the need for many replicates or repetitions. The assay is fast with robust read-outs in 2 weeks. These features provide a major conceptual and technical advance compared with current assays requiring RT-PCR or blotting approaches. While the biology of duck and human cccDNA is similar, it is notable that differences may exist in cccDNA formation between DHBV and HBV, as already suggested by the much higher cccDNA levels DHBV can accumulate in the same cells^8^ and DHBV’s apparently more pronounced dependence on TDP2.^9 26^ However, the identification of known HBV cccDNA-dependency factors beyond TDP2 such as POLK, and FEN1 confirms the ability of our approach to use DHBV cccDNA as a template to identify HBV cccDNA-related host factors. Furthermore, we have developed an HA2/3 analogous HBV cell line with cccDNA dependent HA-HBeAg production (HAC18) in which we could confirm the negative impact of YBX1 depletion (online supplemental figure S2). However, the high cccDNA levels in HA2/3 cells remain a major technical advantage because they also correlate with higher HA-ELISA signals. We note that HA-HBeAg secretion in our assay would respond not only to alterations in the multiple steps of cccDNA formation and/or transcription from the cccDNA minichromosome, but also to factors involved in translation of the precore protein and/or its processing and secretion as HBeAg. Our choice of anti-DDR shRNAs makes the latter scenario unlikely for the current study yet also opens additional future opportunities.

Using this approach, we identified YBX1 as a previously undiscovered HBV host-dependency factor, although not essential for cccDNA production as it is not totally abolished in *YBX1*-KO cells (figure 4). The functional relevance of YBX1 for the HBV life cycle was confirmed by several lines of evidence: (1) Genetic *YBX1* loss-of-function analyses using CRISPR-Cas9 or shRNA resulted in a marked decrease in HBV infection in cell lines and primary human hepatocytes. (2) A substantial reduction in cccDNA levels confirmed functional relevance of YBX1 to synthesise and maintain robust cccDNA levels. (3) A specific recruitment of YBX1 to HBV genome. YBX1 is a multifunctional DNA/RNA-binding protein that regulates transcription and translation.^19^ Increased levels of YBX1 have been correlated with DNA topoisomerase II activity, already described as cccDNA-related HBV host factor. Moreover, a previous study reported a direct interaction between YBX1 and PCNA,^34^ one of the crucial factors required for *in vitro* cccDNA formation.^12^ Although we do not observe a modulation of PCNA expression on *YBX1* silencing (online supplemental figure S5), a detailed understanding of the functional role of YBX1-PCNA interaction in cccDNA formation would require additional studies. Collectively, our data indicate events after entry and preceding establishment of the cccDNA pool as major target(s) for YBX1 proviral activity. However, further studies are needed to unravel in more detail the molecular mechanism(s) of YBX1 in cccDNA formation and/or regulation in the HBV life cycle. YBX1 is a targetable protein and therefore a candidate for antiviral therapy. Small molecules specifically targeting YBX1 should be tested to assess its potential as an antiviral target. Although several YBX1-targeting drugs have been described, such as fisetin^23^ or CYT387,^24^ they have additional targets beyond YBX1.^24 35 36^ YBX1-specific specific inhibitors would be required to further confirm the targetability of YBX1 using a small molecule approach.

Since YBX1 is involved in the modulation of several signal transductions pathways, HBV-YBX1 interaction may also play a role in the pathogenesis of virus-induced liver disease and HCC. Indeed, in patients, *YBX1* expression is accompanied by expression of genes involved in liver disease progression, as well as lower survival of HCC patients. High expression of *YBX1* is often detected in various cancers including HCC, and is closely related to the progression, poor prognosis and multidrug resistance.^37 38^ In this regard it is also of interest to note that YBX1 has been suggested as target in anticancer therapies.^39^ However, additional experiments are required to fully evaluate the functional role of HBV in the regulation of *YBX1* expression and disease biology.

Collectively, the implication of our study is threefold: (1) We have established a robust and simple cell-based system enabling to discover HBV cccDNA-dependency factor as targets for viral cure; (2) our study uncovers the DNA repair factor YBX1 as an important factor for cccDNA biology, providing an opportunity for exploring YBX1 as target for HBV cure; (3) we observed an association between HBV infection, *YBX1* expression and virus-induced liver disease suggesting that targeting YBX1 may not only reduce viral cccDNA but potentially also ameliorate HBV-induced liver disease and cancer incidence.

## Materials and methods

All the materials, reagents and protocols are detailed in online supplemental material.

## Data Availability

Data are available on reasonable request. All data relevant to the study are included in the article or uploaded as online supplemental information.
