# Supplementary material for: Cell-based cccDNA reporter assay combined with functional genomics identifies YBX1 as HBV cccDNA host factor and antiviral candidate target
Source: Gut. 2022 Dec 9;72(9):1745–57. doi: 10.1136/gutjnl-2020-323665 (PMC10423543; doi:10.1136/gutjnl-2020-323665)
Supplement: Supplementary data [file gutjnl-2020-323665supp005.pdf]

A cell-based cccDNA reporter assay combined with functional genomics identifies YBX1 as HBV cccDNA host factor and antiviral candidate target

Eloi R. Verrier[$], Gaëtan Ligat[$], Laura Heydmann, Katharina Dörnbrack, Julija Miller, Anne Maglott-Roth, Frank Jühling, Houssein El Saphira, Margaux J. Heuschkel, Naoto Fujiwara, Sen-Yung Hsieh, Yujin Hoshida, David E. Root, Emanuele Felli, Patrick Pessaux, Atish Mukherji, Laurent Mailly, Catherine Schuster, Laurent Brino, Michael Nassal[&], Thomas F. Baumert[&]

[$]co-first author. [&]co-senior authors.

ONLINE SUPPLEMENTARY MATERIAL

MATERIAL AND METHODS

Human subjects

Primary human hepatocytes (PHH) were obtained from liver tissue from patients undergoing liver resection for liver metastasis at the Strasbourg University Hospitals with informed consent. Protocols were approved by the local Ethics Committee of the Strasbourg University Hospitals (CPP) and the Ministry of Higher Education and Research of France (DC 2016 2616). PHH were isolated and cultured as described [1]. Human samples from HBV infected patients followed at the Chang Gung Memorial Hospital (Taipei, Taiwan) were obtained with informed consent. Protocols were approved by the local Ethics Committee (Institutional Review Board 102-3825C).

Patient and Public Involvement

Not applicable.

Reagents and plasmids

Dimethyl sulfoxide (DMSO), polybrene, Tween-20 and PEG 8000 (polyethylene glycol) were obtained from Sigma-Aldrich (Merck). Paraformaldehyde was obtained from Euromedex. DNA transfection was performed using CalPhos Mammalian Transfection kit (Clontech) according to the manufacturer's instructions. The shRNA-encoding lentivirus constructs were obtained from the RNAi Platform, Broad Institute of MIT and Harvard (Cambridge, USA). pTRE2-hyg plasmid was used to generate a Tet-controlled HepG2 cell line expressing a modified envelope-deficient full-length DHBV genome. Small guide (sg)RNAs were cloned into the Cas9 expressing pLKO puro plasmid (plasmid #8453, Addgene). The expression lentivirus constructs were obtained from Vectorbuilder (VB201221-1030xyb: sgRNA-resistant wild type YBX1 (YBX1-WT); VB190320-1155uyp: control vector). Vector sequences are available online (https://en.vectorbuilder.com/design/retrieve.html). Cell viability was assessed using PrestoBlue™ Cell Viability Reagent (Thermo Fisher).

Cell lines

Human embryonic kidney 293T (HEK 293T) cell line has been described [1]. Purification of infectious HBV particles from the inducible human hepatoblastoma HepAD38 has been described [2, 3]**.** For the production of NTCP-expressing HepG2 and Huh7 cells, cells were seeded in six-well plates at 50% confluence 1 day prior to transduction with human NTCP-expressing vesicular stomatitis virus pseudoparticles (pp) (GeneCopoeia). After 3 days, cells were expanded and selected for NTCP expression with 500 µg/mL of neomycin (G418). HepG2-NTCP and Huh7-NTCP cells were maintained at a concentration of 250 µg/mL G418.

Verrier ER, *et al. Gut* 2023; 72:1745–1757. doi: 10.1136/gutjnl-2020-323665

HBV and HDV production

The production of recombinant HBV (ayw) and HDV (genotype 1) infectious virus have been described [3, 4]. The primers used for quantification of HBV RNA (both pgRNA and preC RNA) and HDV RNA by RT-qPCR following infection are indicated in Supplementary Table S3.

Establishment of a Tet-controlled HepG2 cell line expressing a modified full-length DHBV genome

DHBV-HA2/3env$^-$ (HA2/3 in brief) is a clonal cell line based on the human HepG2 hepatoma cell line derivative HepG2.TA2-7 that stably expresses the Tet-transactivator tTA2, maintained by G418 selection [5], and a derivative of the TRE promoter plasmid pTRE2-hyg which carries the hygromycin resistance gene. Into this plasmid a 1.1x unit lengths DHBV16 genome (GenBank K01834) carrying a a stop mutation in the preS region (G1165A) and a HA-tag (YPYDVPDYA) coding sequence in the preC region four codons upstream (DpC_HA1) or immediately (DpC_HA2) in front of the core ORF was inserted such that TRE promoter initiates transcription at the authentic pgRNA start site. Of the resulting plasmids pTRE-DHBVpC_HA2-env$^-$ was transfected into HepG2.TA2-7 cells and stable cell clones were selected via hygromycin. Clone HA2/3 showed robust, Dox withdrawal-dependent replication initiation, cccDNA formation and HAeAg secretion. To access capsid-associated and nuclear viral DNAs, cytoplasm and nuclei were separated by NP40 lysis, then nucleocapsid-associated and nuclear DNAs were extracted and detected by Southern blotting using a ^{32}P labeled DNA probe as previously described [6], except that to the nuclei a constant amount of the 6.3 kb DHBV16 expression plasmid pCD16 was added to monitor the subsequent DNA extraction [6]. HAC18 cells were produced using the same strategy replacing DHBV by HBV genome.

Detection of DHBeAg by specific anti-HA ELISA and Immunofluorescence

HA2/3 cells were seeded in 96-well plates and doxycycline was withdrawn to allow pgRNA transcription. Supernatants were harvested at different time points for the specific detection of HA-taged DHBeAg (HA-DHBeAg) through anti-HA sandwich ELISA. 96-well microplates (Thermo Fisher Scientific) were pre-coated with anti-DHBcAg chicken Ba09 antibody at a dilution of 1:1000 overnight at 4 °C. After blocking with PBS, 10% FBS, Tween 20 (0,05%) for 3 h, the plates were washed with PBS containing 0.05% Tween 20 four times. 40 µL of each sample were incubated in the coated plates overnight at 4°C. The plates were washed with PBS containing 0.05% Tween 20 four times and incubated with mouse anti-HA-PO antibody (3F10, Roche) at a dilution of 1:1000 for 1 h. Plates were washed samples were incubated with peroxidase-conjugated, goat anti-mouse-PO IgG at a dilution of 1:5000 (NA931, GE Healthcare). After washing, the HA-DHBeAg levels were revealed with the SuperSignal™ ELISA Femto Substrate (Thermo Fisher Scientific) for 1 min. Chemiluminescence was determined at 425 nm using a Mithras LB940 Microplate reader (Berthold). For Immunofluorescence detection of DHBcAg and DHBeAg, HA2/3 cells were seeded in 96-well plates and doxycycline was withdrawn to allow pgRNA transcription. Cells were fixed with 4% paraformaldehyde (PFA). DHBcAg and HA-DHBeAg were detected using a specific mouse antibody (21-5-10c) and a specific mouse anti-HA tag antibody (HA tag antibody [HA.C5] (ab18181), respectively, and an AF647-labelled secondary antibody targeting mouse IgGs (Jackson Research). Cell nuclei were stained with DAPI. Fluorescent imaging was performed using an Axio Vert.A1 microscope (Carl Zeiss).

Targeted loss-of-function screen using a shRNA-encoding lentiviral expression library

A DNA-repair targeting shRNA library was designed and prepared at the RNAi platform from the Broad Institute of MIT and Harvard, Cambridge, USA. The 239 targeted genes are listed in Supplementary Table S1. Three shRNA constructs per gene were designed. shRNA constructs were cloned into a pLKO vector carrying the puromycin resistance gene HA2/3 cells, initially cultured in the presence of doxycycline, were plated in 96-well plates, and cultured for one day in doxycycline-free medium prior to transduction, in triplicate, with the shRNA-encoding lentiviral expression library. Cells were selected with puromycin (2 µg/ml) three days after transduction. At 21 days post platting and doxycycline withdrawal, the supernatants were

Verrier ER, *et al. Gut* 2023; 72:1745–1757. doi: 10.1136/gutjnl-2020-323665

harvested for quantification of secreted HA-DHBeAg as described above. Stable and long-term shRNA-induced gene expression silencing was assessed by the quantification of YBX1 expression, one candidate of the screen, at days 10 and 21 post plating (Supplementary Figure S1). The global effect of individual gene silencing on HA-DHBeAg production was analyzed by pooling the effect of the 3-independent shRNA per gene (n=9). *p* values were obtained through a Student's t-test comparing phenotypic effects of shRNAs and pLKO ctrl. The analysis of the candidates included phenotypic robustness between replicates, toxicity (PrestoBlue™ > 0.75 compared to pLKO ctrl), and expression in the liver (Illumina Body Map).

Loss-of-function studies in HBV/HDV permissive cells and primary human hepatocytes
Individual shRNA-encoding lentiviral particles were produced in HEK 293T cells by cotransfection of plasmids expressing the human immunodeficiency virus (HIV) gag-pol, the vesicular stomatitis virus glycoprotein (VSV-G) and the pLKO.1 puro-shRNA plasmids in the ratio of 10:3:10, using the CalPhos Mammalian Transfection kit as described [1]. Three days after transfection, supernatants were collected and clarified using 0.45 µm pore filters. HepG2-NTCP were transduced with shRNA-encoding lentivirus and selected with puromycin 2 µg/mL 48 hours prior to HBV infection. Alternatively, PHH were transduced with individual shRNA-containing lentivirus prior to HBV infection. To generate YBX1-KO HepG2-NTCP and Huh7-NTCP cell lines, the following primers corresponding to guide RNAs targeting YBX1 exons were cloned into the generated Cas9 expressing pLKO puro plasmid (plasmid #8453, Addgene): Forward primer: CACCGACGGATATGGTTTCATCAAC, Reverse primer: AAACGTTGATGAAACCATATCCGTC. For robust KO early stage passages of cells need to be used. For rescue experiments, YBX1-KO HepG2-NTCP were transduced with ctrl vector, or YBX1-WT-containing lentivirus constructs and selected with puromycin 48 hours prior to HBV infection. HepG2-NTCP and PHH were infected by HBV (GEq 500 per cell and 1500 per cell, respectively) as described [7]. HBV infection was assessed 10 days post infection (dpi) by quantification of HBeAg using chemiluminescence immunoassay (CLIA, Autobio) following the manufacturer's instructions. Alternatively, cells were lysed and total RNA was extracted using the ReliaPrep RNA Miniprep Systems (Promega) and qRT-PCR quantification of HBV pgRNA was performed as described [3]. Alternatively, cells were fixed with 4% PFA. HBsAg was immunodetected using a specific mouse monoclonal anti-HBsAg antibody (clone 1044/329, Bio-Techne, USA) and Alexa Fluor 647-labelled secondary antibody targeting mouse IgGs (Jackson Research). Cell nuclei were stained with DAPI. Fluorescent imaging was performed using an Axio Vert.A1 microscope (Carl Zeiss). In addition, YBX1-KO Huh7-NTCP cells were produced as described above for YBX1-KO HepG2-NTCP cells and infected with HDV. HDV infection was assessed 7 dpi by qRT-PCR quantification of HDV RNA or immunodetection of HDAg as described [4]. Fluorescent imaging was performed using an Axio Vert.A1 microscope (Carl Zeiss).

cccDNA detection by Southern blot and qPCR.
Southern blot detection of HBV cccDNA was performed using digoxigenin (DIG)-labeled (Roche) specific probes as described [8]. Total DNA from HBV-infected cells was extracted using the Hirt method as described [9]. Specific DIG-labeled probes for the detection of HBV and mitochondrial DNAs were synthetized using the PCR DIG Probe Synthesis Kit (Roche) and the primers as described [7]. DNA Molecular Weight Marker II and VII (Roche) were used. cccDNA levels were quantified using ImageJ software.
Specific cccDNA qPCR method was already described [8, 10]. DNA was extracted with a protocol adapted from the MasterPureTM DNA Purification Kit (Epicentre).
The following primers and probes were used:
cccDNA Taqman Probe 1864-1882: [6FAM]CATGGAGACCACCGTGAACGCCC[BHQ1] (final concentration: 0.2µM) ;
FW Primer: CCGTGTGCACTTCGCTTCA (final concentration: 0.1µM)
RV Primer: GCACAGCTTGGAGGCTTGA (final concentration: 0.8µM).
cccDNA levels were normalized to βglobin levels (TaqMan® Gene Expression Assay, Hs00758889_s1, Life Technologies).

Analysis of gene expression by RT-qPCR

RNA was extracted as described above and gene expression was assessed by qRT-PCR as described [7]. Gene expression was normalized to GADPH expression. Primers and TaqMan® probes for POLK (Hs00211965_m1), TDP2 (Hs01099017_m1), YBX1 (Hs00358903_g1) and PCNA (Hs00427214_g1) mRNA quantification were obtained from ThermoFisher (TaqMan® Gene Expression Assays, Life Technologies).

Analysis of protein expression by Western blot

The protein expression of YBX1, phospho-YBX1 (S102), GAPDH and β-actin was assessed by Western blot as described [7] using a monoclonal rabbit anti-YBX1 antibody (ab76149, Abcam), a polyclonal rabbit anti-YBX1(phospho S102) antibody (ab138654, Abcam) a polyclonal rabbit anti-GAPDH (ab9485, Abcam), and a monoclonal mouse anti-β-actin (A5441, Sigma), respectively. Protein expression was assessed using the ChemiDocTM Imaging System (Bio-Rad).

Prediction of a putative Y-box motif within HBV genome

The HBV genome sequence of reference strain ayw and adw was aligned with the Y-box consensus sequence. Alignments were performed with Clustal Omega (Ω) multiple sequence alignment (MSA) tool provided by the EMBL-EBI bioinformatics web and programmatic tools framework [11].

ChIP-qPCR Assay

HepG2-NTCP were infected by HBV (GEq 500 per cell) as described [7]. The ChIP protocol was adapted from [12] with few minor modifications. Two days post infection, fresh formaldehyde was added [0.4% final concentration (v/v)] to the PBS containing cells and incubated on a flip-flop rocker for 10 min at room temperature, followed by addition of 2M glycine (0.125M final concentration) quenched with 0.25 M glycine for 5 min, and mechanically lysed at 4°C in lysis buffer (PIPES 5 mM, KCL 85 mM, NP-40 0.5%), with protease inhibitors (Roche). After low-speed centrifugation, nuclei were lysed in SDS-buffer (EDTA 10 mM, Tris-HCl pH8 50 mM, SDS 1%). After sonication (Covaris), clarified lysates were diluted in RIPA-buffer (Tris-HCl pH-7.5 10 mM, NaCl 140 mM, EDTA 1 mM, EGTA 0.5 mM, 1% Triton-X100, 0.1% SDS, 0.1% Na-Deoxycholate). Nuclear lysates were precleared with magnetic protein G-beads (10004D, Invitrogen) before immunoprecipitation with rabbit IgG isotype control (ChIP grade, ab76149, Abcam), a rabbit anti-YBX1 antibody (ab76149, Abcam) or an anti-HBcAg antibody (MA1-7607, Invitrogen) at 4°C. Magnetic beads were added for 2 h to capture immune complexes. Flow-through fractions were collected, and beads washed with RIPA-buffer. Immunoprecipitated complexes were either boiled in Laemmli 1X for western blot analysis or washed with TE (Tris-HCl pH-8 10 mM, EDTA 10 mM) and then incubated for 2 h at 68°C in elution buffer (Tris-HCl pH-7.5 20 mM; EDTA 5 mM; NaCl 50 mM; 1% SDS; Proteinase-K 50 µg/ml). DNA was then extracted and quantified by qPCR using specific primers (TaqMan® Gene Expression Assay, HBV, ID Pa03453406, Life Technologies) as described [12, 13]. DNA was similarly purified from the flow-through fraction and used as input for calculations. Protein expression in each sample was assessed by Western blot using a a rabbit anti-YBX1 antibody (ab76149, Abcam) or an anti-HBcAg antibody (MA1-7607, Invitrogen), and VeriBlot for IP Detection Reagent (HRP) (ab131366, Abcam).

Infection of FRG-NOD mice for ChIP-qPCR assay.

We used HBV-infected FRG-NOD mice from the cohort of a previously published study [14]. Mice were housed and bred at the INSERM U1110 animal facility (regional agreement n°E-67-482-7) and fed 2-(2-nitro-4-trifluoromethylbenzoyl)-1,3-cyclohexanedione (NTBC) in their drinking water (16 mg/L). Six-week-old FRG-NOD mice received 1.5 × 10^9 pfu of an adenoviral vector encoding for the urokinase-like plasminogen activator, and treated with NTBC (8 mg/L). Forty eight hours later mice were intrasplenically transplanted with 10^6 PHHs (Corning) and given NTBC at 0.8 mg/L. During the following days NTBC dose was decreased every 2 days to 0.4 mg/L and 0.2 mg/L, and finally withdrawn. Efficient transplantation was assessed 8

weeks later by measuring human serum albumin levels by ELISA (Bethyl). The transplantation procedure was approved by the local Ethics committee and authorized by the French ministry of research and higher education (APAFIS#4485-2016031115352125 v3). Successfully transplanted mice were infected with 10^9 HBV (ayw) genome equivalents. The experimental procedure was approved by the local Ethics committee and authorized by the French ministry of research and higher education (APAFIS#13872-2018050214497349 v1). Human albumin levels were determined as described previously [15]. Mice were sacrificed and livers harvested after 10 weeks of HBV infection. 3 "control" mice from the [14] study were used. HBV DNA viral load was determined at the time of sacrifice using the clinically approved Abbott Real Time HBV assay (Abbott). Human albumin levels were determined as described previously [15]. Viral loads and albumin levels per mouse are presented in Supplementary Table S4. Liver samples were lyzed and ChIP-qPCR assay for HBV DNA quantification upon immunoprecipitation using a specific anti-YBX1 antibody was performed as described above. The only difference is the use a Bioruptor (Diagenode) device for the sonication of samples.

Analysis of YBX1 expression in patients.

For the analysis of YBX1 mRNA expression in patients, total RNA was extracted from liver tissue of 9 HBV-infected patients by using High Pure RNA Paraffin kit (Roche) according to the manufacturer's instruction, and gene expression analysis was performed by RNA-seq as previously reported [16]. To analyze the correlation between YBX1 expression and the progression of liver disease in HBV-infected patients, YBX1 mRNA expression was assessed in HBV-related patients from GSE121248 [17]. For survival and recurrence analysis, data of HBV-induced HCC patients were derived from GSE14322 [18]. To analyze the correlation between YBX1 expression and the NASH fibrosis stage, YBX1 mRNA expression was assessed in HBV-related patients from GSE49541 [19]. For survival and HCC development analysis, data of HCV-induced HCC patients were derived from GSE14322 [20].

Statistical analyses

Statistical analyses were performed using a two-tailed Mann-Whitney U test unless otherwise stated; $p < 0.05$ (★), $p < 0.01$ (★★), and $p < 0.001$ (★★★) were considered statistically significant. Correlation between YBX1 expression and HBV viral load in patients was assessed using Spearman's rank correlation coefficient (Spearman's rho). Survival functions depending on YBX1 expression were obtained using the Kaplan-Meier estimator. *p*-value was calculated using log-rank test for comparisons of Kaplan-Meier survival. $p < 0.05$ was considered statistically significant. Significant *p*-values are indicated by asterisks in the individual figures and figure legends.

Verrier ER, *et al. Gut* 2023; 72:1745–1757. doi: 10.1136/gutjnl-2020-323665

SUPPLEMENTARY FIGURES

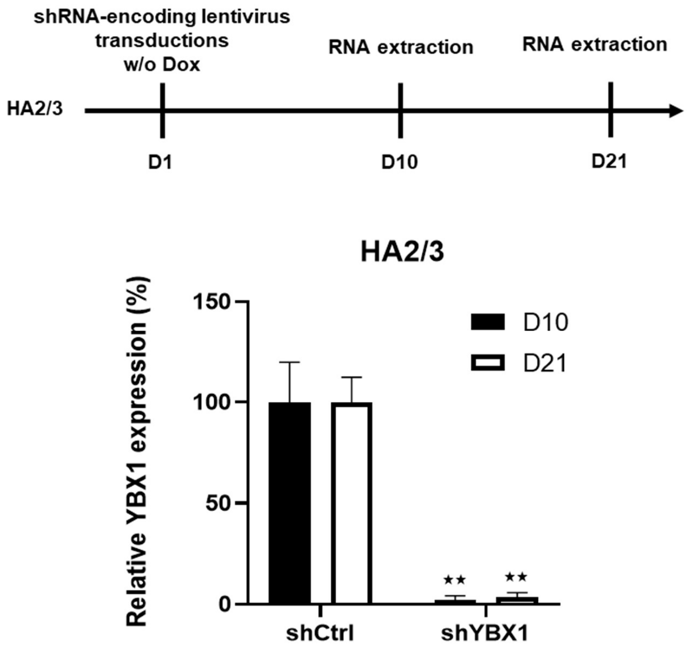

Figure S1: Control of long-term silencing of YBX1 expression in HA2/3 cells. HA2/3 cells were plated in 96well-plates one day prior to transduction. YBX1 expression was assessed by qRT-PCR at day 10 and day 21 post platting. Results are expressed as means ± SD % relative YBX1 expression compared to shCtrl (set at 100%) from 3 independent experiments. ★★ $p < 0{,}01$ (two-tailed Mann–Whitney U test).

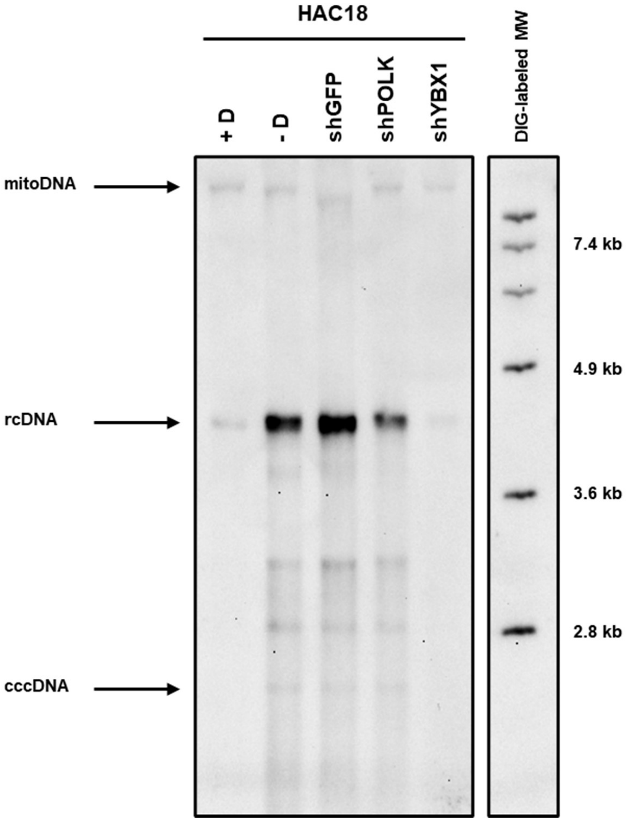

Figure S2: POLK- and YBX1 specific knock-down decreases cccDNA levels in HAC18 cells. HAC18 were transduced with shRNA-encoding lentivirus, without Dox and selected with puromycin 2 µg/mL 48 hours. Detection of HBV DNAs by Southern Blot 21 days post platting. HBV rcDNA and HBV cccDNA are indicated.

Verrier ER, *et al. Gut* 2023; 72:1745–1757. doi: 10.1136/gutjnl-2020-323665

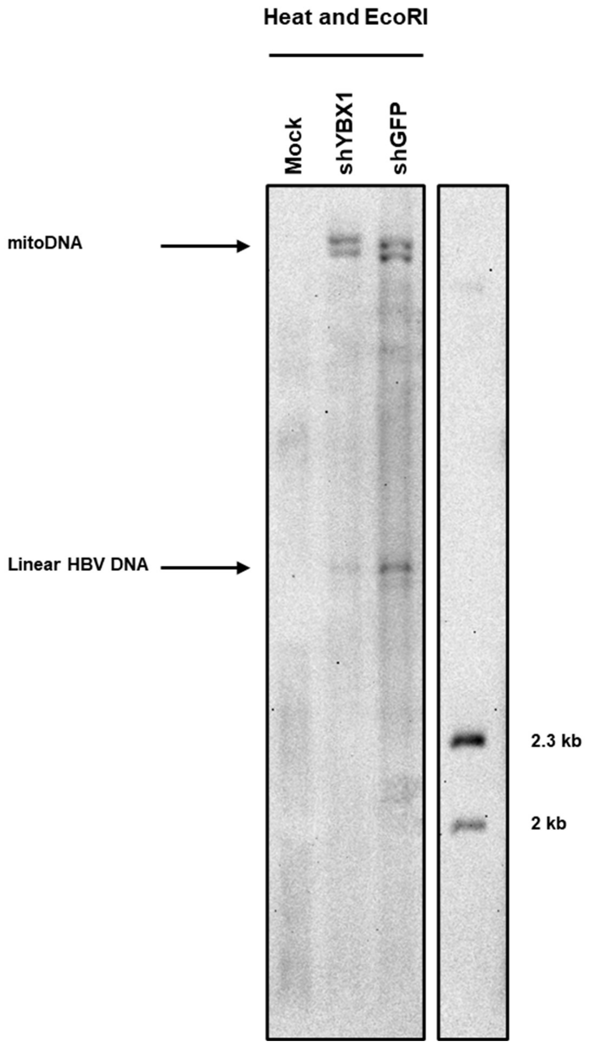

Figure S3: Linearization of HBV cccDNA. HBV DNAs were submitted to heat treatment (85°C) and subsequent EcoRI digestion (control related to the Figure 4J).

Verrier ER, *et al. Gut* 2023; 72:1745–1757. doi: 10.1136/gutjnl-2020-323665

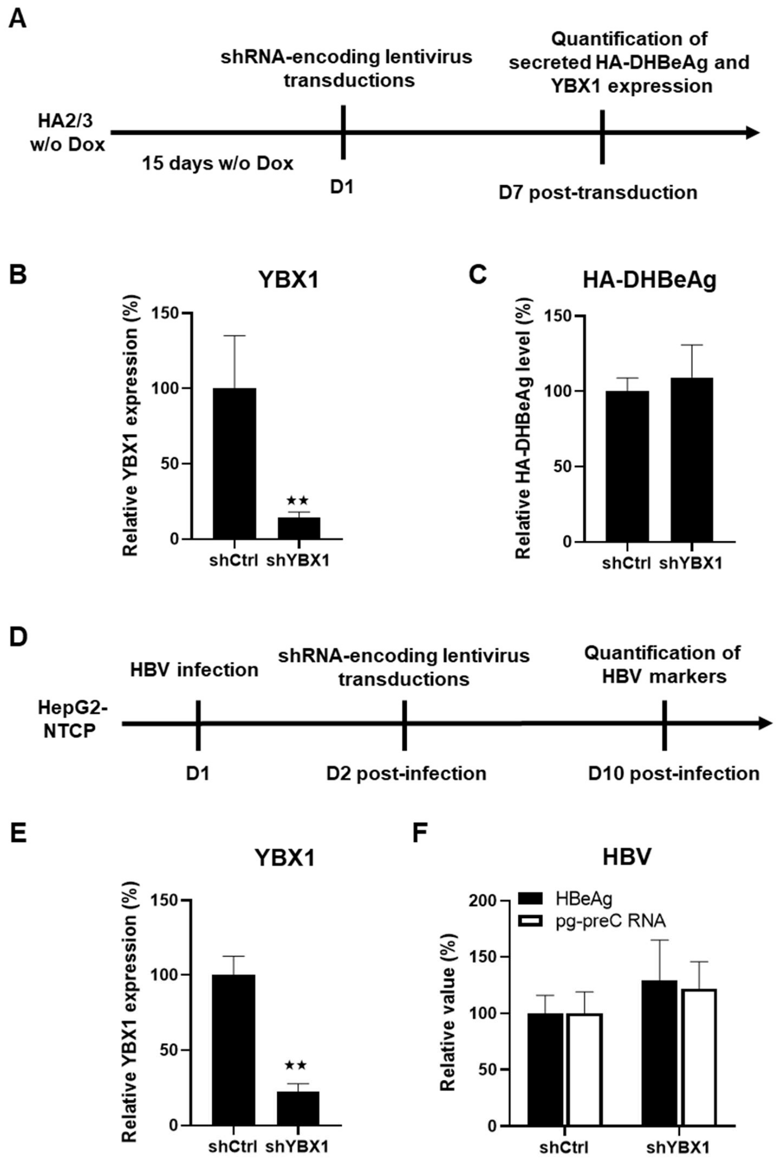

Figure S4: YBX1 knock-down after establishment of the cccDNA pool affects HBV replication neither in HA2/3 cells nor in infected HepG2-NTCP cells. A. Experimental timelines. **B.** YBX1 mRNA expression upon silencing, assessed by qRT-PCR. Results are expressed as means ± SD % relative YBX1 expression compared to shCtrl (set at 100%) from three independent experiments. **C.** HA-DHBeAg production quantified by anti-HA ELISA 7 days after transduction. Results are expressed as means ± SD % relative HA-HBeAg secretion compared to shCtrl (set at 100%) from three independent experiments. **D.** Experimental timelines. **E.** YBX1 mRNA expression upon silencing; assessed by qRT-PCR. Results are expressed as means ± SD % relative YBX1 expression compared to shCtrl (set at 100%) from three independent experiments. **F.** Detection of HBV markers 10 dpi: HBeAg is quantified by CLIA (black), HBV pg-preC RNA is quantified by qRT-PCR (white). Results are expressed as means ± SD % relative HBV infection compared to shCtrl (set as 100%) from 3 independent experiments. ★★ $p < 0,01$ (two-tailed Mann–Whitney U test).

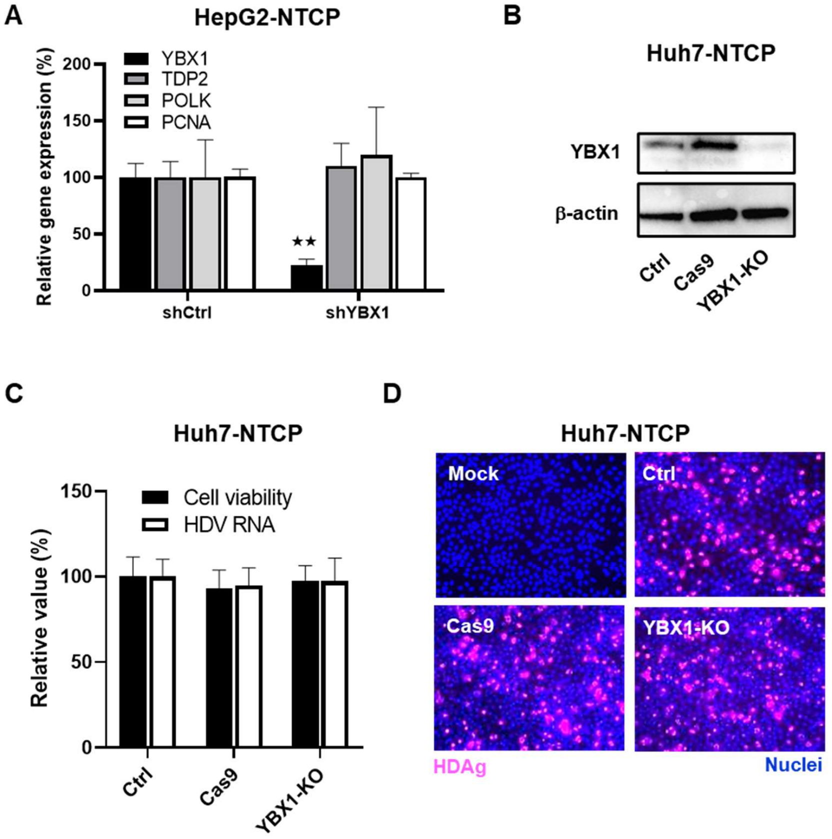

Figure S5. Impact of YBX1 expression on the early steps of the HBV life cycle. A. YBX1 silencing does not modulate the expression of cccDNA-related factors. YBX1 expression was silenced in HepG2-NTCP cells using specific shRNA. Silencing efficacy was assessed by YBX1 mRNA quantification by qRT-PCR. Samples from Figure S4E were used. The expression of YBX1, POLK, TDP2, and PCNA was analyzed by qRT-PCR. Results are expressed as means ± SD % relative expression compared to shCtrl (set at 100%) from 3 independent experiments. **B-D.** No effect of YBX1 KO on HDV infection in Huh7-NTCP cells. YBX1 expression was controlled by Western blot in Huh7-NTCP (ctrl), cas9-expressing Huh7-NTCP cells (cas9), and YBX1-KO Huh7-NTCP (**B**). Cells were then infected by HDV for 7 days. Cell viability was assessed 7 dpi. Results are expressed as means ± SD % relative cell viability compared to HDV-infected Huh7-NTCP (set at 100%) from 2 independent experiments (black) In parallel, total RNA was extracted and HDV infection was assessed by quantification of HDV RNA by RT-qPCR (white) (**C**). Results are expressed as means ± SD % relative HDV infection compared to HDV-infected Huh7-NTCP (set as 100%) from 2 independent experiments. Alternatively, intracellular HDAg levels were assessed by IF 7 dpi (**D**). ★★ $p < 0,01$ (two-tailed Mann–Whitney U test).

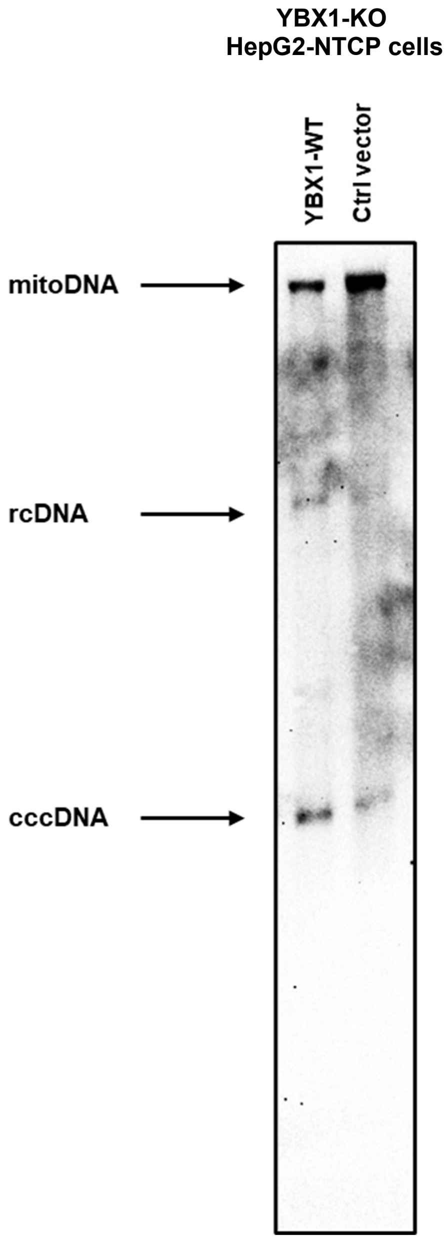

Figure S6: Expression of YBX1 increases cccDNA levels in YBX1-KO cells. YBX1-KO HepG2-NTCP cells were transduced with lentivirus encoding the wild type version of YBX1 (YBX1-WT) or an empty vector (control). 6 days after transduction, cells were infected by HBV. 2 days post infection, DNA was extracted and HBV DNA was detected by Southern blot.

Verrier ER, *et al. Gut* 2023; 72:1745–1757. doi: 10.1136/gutjnl-2020-323665

SUPPLEMENTARY REFERENCES

1. Lupberger J, Zeisel MB, Xiao F, et al. EGFR and EphA2 are host factors for hepatitis C virus entry and possible targets for antiviral therapy. *Nat Med*. 2011;17(5):589-95.

2. Ladner SK, Otto MJ, Barker CS, et al. Inducible expression of human hepatitis B virus (HBV) in stably transfected hepatoblastoma cells: a novel system for screening potential inhibitors of HBV replication. *Antimicrob Agents Chemother*. 1997;41(8):1715-20.

3. Verrier ER, Colpitts CC, Bach C, et al. A targeted functional RNA interference screen uncovers glypican 5 as an entry factor for hepatitis B and D viruses. *Hepatology*. 2016;63(1):35-48.

4. Verrier ER, Weiss A, Bach C, et al. Combined small molecule and loss-of-function screen uncovers estrogen receptor alpha and CAD as host factors for HDV infection and antiviral targets. *Gut*. 2020;69(1):158-67.

5. Schreiner S, Nassal M. A Role for the Host DNA Damage Response in Hepatitis B Virus cccDNA Formation-and Beyond? *Viruses*. 2017;9(5).

6. Kock J, Rosler C, Zhang JJ, et al. Generation of covalently closed circular DNA of hepatitis B viruses via intracellular recycling is regulated in a virus specific manner. *PLoS Pathog*. 2010;6(9):e1001082.

7. Verrier ER, Yim SA, Heydmann L, et al. Hepatitis B Virus Evasion From Cyclic Guanosine Monophosphate-Adenosine Monophosphate Synthase Sensing in Human Hepatocytes. *Hepatology*. 2018;68(5):1695-709.

8. Lucifora J, Salvetti A, Marniquet X, et al. Detection of the hepatitis B virus (HBV) covalently-closed-circular DNA (cccDNA) in mice transduced with a recombinant AAV-HBV vector. *Antiviral Res*. 2017;145:14-9.

9. Gao W, Hu J. Formation of hepatitis B virus covalently closed circular DNA: removal of genome-linked protein. *J Virol*. 2007;81(12):6164-74.

10. Allweiss L, Volz T, Giersch K, et al. Proliferation of primary human hepatocytes and prevention of hepatitis B virus reinfection efficiently deplete nuclear cccDNA in vivo. *Gut*. 2018;67(3):542-52.

11. Sievers F, Wilm A, Dineen D, et al. Fast, scalable generation of high-quality protein multiple sequence alignments using Clustal Omega. *Mol Syst Biol*. 2011;7:539.

12. Lucifora J, Pastor F, Charles E, et al. Evidence for long-term association of virion-delivered HBV core protein with cccDNA independently of viral protein production. *JHEP Rep*. 2021;3(5):100330.

13. Lucifora J, Bonnin M, Aillot L, et al. Direct antiviral properties of TLR ligands against HBV replication in immune-competent hepatocytes. *Sci Rep*. 2018;8(1):5390.

14. Zhuang X, Forde D, Tsukuda S, et al. Circadian control of hepatitis B virus replication. *Nat Commun*. 2021;12(1):1658.

15. Mailly L, Xiao F, Lupberger J, et al. Clearance of persistent hepatitis C virus infection in humanized mice using a claudin-1-targeting monoclonal antibody. *Nat Biotechnol*. 2015;33(5):549-54.

Verrier ER, *et al. Gut* 2023; 72:1745–1757. doi: 10.1136/gutjnl-2020-323665

16. Nakagawa S, Wei L, Song WM, et al. Molecular Liver Cancer Prevention in Cirrhosis by Organ Transcriptome Analysis and Lysophosphatidic Acid Pathway Inhibition. *Cancer Cell*. 2016;30(6):879-90.

17. Wang SM, Ooi LL, Hui KM. Identification and validation of a novel gene signature associated with the recurrence of human hepatocellular carcinoma. *Clin Cancer Res*. 2007;13(21):6275-83.

18. Roessler S, Long EL, Budhu A, et al. Integrative genomic identification of genes on 8p associated with hepatocellular carcinoma progression and patient survival. *Gastroenterology*. 2012;142(4):957-66 e12.

19. Murphy SK, Yang H, Moylan CA, et al. Relationship between methylome and transcriptome in patients with nonalcoholic fatty liver disease. *Gastroenterology*. 2013;145(5):1076-87.

20. Hoshida Y, Villanueva A, Sangiovanni A, et al. Prognostic gene expression signature for patients with hepatitis C-related early-stage cirrhosis. *Gastroenterology*. 2013;144(5):1024-30.
